# The subcellular redistribution of NLRC5 promotes angiogenesis via interacting with STAT3 in endothelial cells

**DOI:** 10.7150/thno.54473

**Published:** 2021-03-04

**Authors:** Xu Xu, Yefei Shi, Peipei Luan, Wenxin Kou, Bo Li, Ming Zhai, Shuangjie You, Qing Yu, Jianhui Zhuang, Weixia Jian, Mark W. Feinberg, Wenhui Peng

**Affiliations:** 1 Department of Cardiology, Shanghai Tenth People's Hospital, Tongji University School of Medicine, Shanghai, China.; 2 Department of Endocrinology, Xinhua Hospital, Shanghai Jiaotong University, School of Medicine, Shanghai, China.; 3 Cardiovascular Division, Department of Medicine, Brigham and Women's Hospital, Harvard Medical School, Boston, Massachusetts, USA.

**Keywords:** angiogenesis, NLRC5, STAT3, signal transduction, endothelial cell

## Abstract

Angiogenesis is a critical step in repair of tissue injury. The pattern recognition receptors (PRRs) recognize pathogen and damage associated molecular patterns (DAMPs) during injury and achieve host defense directly. However, the role of NLR family CARD domain containing 5 (NLRC5), an important member of PPRs, beyond host defense in angiogenesis during tissue repair remains unknown.

**Methods:** *In vitro,* western blot and real-time PCR (RT-PCR) were used to detect the expression of NLRC5 in endothelial cells (ECs). Immunofluorescence microscopy was used to reveal the subcellular location of NLRC5 in ECs. Cell proliferation, wound healing, tube formation assays of ECs were performed to study the role of NLRC5 in angiogenesis. By using Tie2Cre-NLRC5^flox/flox^ mice and bone marrow transplantation studies, we defined an EC-specific role for NLRC5 in angiogenesis. Mechanistically, co-immunoprecipitation studies and RNA sequencing indicated that signal transducer and activator of transcription 3 (STAT3) was the target of NLRC5 in the nucleus. And Co-IP was used to verify the specific domain of NLRC5 binding with STAT3. ChIP assay determined the genes regulated by interaction of STAT3 and NLRC5.

**Results:** Knockdown of NLRC5 *in vitro* or *in vivo* inhibited pathological angiogenesis, but had no effect on physiological angiogenesis. NLRC5 was also identified to bind to STAT3 in the nucleus required the integrated death-domain and nucleotide-binding domain (DD+NACHT domain) of NLRC5. And the interaction of STAT3 and NLRC5 could enhance the transcription of angiopoietin-2 (Ang2) and cyclin D1 (CCND1) to participate in angiogenesis.

**Conclusions:** In the ischemic microenvironment, NLRC5 protein accumulates in the nucleus of ECs and enhances STAT3 transcriptional activity for angiogenesis. These findings establish NLRC5 as a novel modulator of VEGFA signaling, providing a new target for angiogenic therapy to foster tissue regeneration.

## Introduction

Pathogen products termed pathogen associated molecular patterns (PAMPs e.g., lipopolysaccharide, proteoglycan, viral RNA) and nuclear or cytosolic molecules termed damage associated molecular patterns (DAMPs e.g., HMGB-1, ATP, DNA) are the two main triggers of inflammation 1, 2. The pattern recognition receptors (PRRs) in host cells recognize the PAMPs or DAMPs and facilitate host defense directly. PPRs are widely studied for their roles in inflammation; yet, their true roles in other disease processes remain poorly defined 2-4. Recently, several PRRs have been found to directly regulate angiogenesis 5, 6. As PRRs are extensively expressed in ischemic and inflammatory microenvironments, it is reasonable to hypothesize that PRRs would not only be involved in tissue injury, but also tissue repair, a process that involves pathological neovascularization.

PPRs have several subfamilies including Toll like receptors (TLRs), Retinoic acid-inducible gene-I-like receptors (RLRs), C-type lectin receptors (CLRs), and the NOD-like receptors (NLRs). NLR family CARD domain containing 5 (NLRC5), is the longest member of the NLRs family 7. It contains an atypical caspase recruitment domain (CARD domain), a central nucleotide-binding domain (NBD domain) and leucine-rich repeats domain (LRRs domain) 8. The small nuclear localization signal ahead of the NBD domain determines the subcellular distribution of NLRC5. The structure characteristics of NLRC5 make its function similar to major histocompatibility class II (MHC II) transactivator (CIITA), another member of NLRs 7, 9. As CIITA has been recognized to regulate MHC II related genes 10-12, NLRC5 regulates the MHC I gene in an analogous manner by forming a complex in the SXY (containing of W/S, X1, X2 and Y boxes) module of the MHC I promoter. Therefore, NLRC5 is also called major histocompatibility class I transactivator (CITA) 13, 14. Early studies demonstrated that NLRC5 inhibits the NF-κB signaling pathway 15-17 and regulates the secretion pro-inflammatory cytokines such as interleukin-6 (IL-6), tumor necrosis factor α (TNFα), and interleukin-1β (IL-1β) both *in vitro* and *in vivo* 7, 16, 17. Moreover, NLRC5 impacts the formation of the NACHT, LRR and PYD domains-containing protein 3 inflammasome 18, 19. Although the inflammatory role of NLRC5 is correlated with its location in the cytoplasm, there remains uncertainty about the nuclear function of NLRC5 beyond classical MHC I regulation. Our group reported that NLRC5 not only modulated the TGFβ signaling pathway, but also regulated the phenotype of vascular smooth muscle cells by directly binding to peroxisome proliferator activated receptor γ (PPARγ) in the nucleus 20, 21. In addition to vascular remodeling 21, we also found that the expression of NLRC5 was regulated in the vascular endothelium. Based on these observations, we hypothesize that NLRC5 may also play an essential role in endothelial cells (ECs).

Angiogenesis is the process that sprouting or intussusceptive ECs form new blood vessels from preexistent ones 22, 23. It exists as early as the embryonic development stage in response to proangiogenic gradients. Such angiogenesis during development is defined as physiological angiogenesis 24. In contrast, pathological angiogenesis, such as angiogenesis involved in ischemia or cancer, is a distinct type due to its microenvironment being infiltrated with PAMPs, DAMPs, and inflammatory cells 25-28. In general, pathological angiogenesis, a rapid, partial, and restricted form of angiogenesis 29, assists injured tissues in cellular clearance and tissue regeneration 27, 30, 31. However, the initiating events enabling heterogeneous ECs to participate in pathological angiogenesis remains poorly defined.

In this study, we identify that NLRC5 translocation to the nucleus of ECs will promote pathological angiogenesis, serving as an activator for STAT3 and STAT3-related genes involved in angiogenesis.

## Methods

### Human sample collection and ethical approval

Human lung tissue sample was obtained from patients undergoing trauma surgery. Human saphenous vein and human internal mammary artery were obtained from patients undergoing coronary artery bypass grafting. The study received approval by the Ethical Committee of Shanghai Tenth People's Hospital and experiments were conducted in compliance with all relevant ethical regulations. The written informed consent was collected from each patient and/or their relatives.

### Cell culture and stimulation

Human umbilical vein endothelial cells (HUVECs) were purchased from Sciencell Research Laboratories (Cat# 8000, Sciencell) and passage 2 to 8 were used for experiments. Cells were cultured in endothelial cells medium (Cat# 1001, Sciencell) with 5% fetal calf serum (FBS), 1% endothelial cells growth supplement (ECGS), 1% penicillin and streptomycin. HUVECs were starved for 4 h, then the medium was supplied with VEGFA-165 (50 ng/mL, Cat# 100-20, PeproTech) or lipopolysaccharide (LPS, 100 ng/mL, Cat# L3024, Sigma-Aldrich) for another 6, 12, 24 h. After stimulations, HUVECs were harvested for other experiments. Human aortic smooth muscle cells (HASMCs) were purchased from Sciencell Research Laboratories (Cat# 6110, Sciencell), and passage 3 to 8 were used for the experiments. Cells were cultured in the smooth muscle cells medium (Cat# 1101, Sciencell) containing 2% FBS, 1% smooth muscle cells growth supplement and 1% penicillin and streptomycin. Human mononuclear cells (THP-1) and human embryonic kidney 293T (HEK293T) cells were obtained from Fudan University Institutes of Biomedical Sciences Cell Center (Shanghai, China) and EA.hy926 cells were purchased from Shanghai Zhongqiaoxinzhou Biotechnology (Cat# ZQ0079). They were cultured in DMEM supplemented with 10% FBS and 1% penicillin and 1% streptomycin.

### Mouse lung endothelial cells isolation and culture

7-10 days old newborn mice were anesthetized with 2% isoflurane for the isolation of lung endothelial cells. Lungs were quickly dissected and cut into small pieces (1 mm or smaller) and digested with Hank's balanced salt solution (HBSS) containing 1 mg/mL collagenase I (Cat# 1148089, Sigma-Aldrich) for 40 min at 37 °C. The mixture of tissues and cells were filtered using sterile 0.45 µm nylon mesh and washed in HBSS containing 5% FBS. The filtered cell suspension was then incubated with Dynabeads (Cat# 557355, Invitrogen) at 4 °C for 30 min to capture ECs. The beads were first coated with anti-mouse CD31 monoclonal antibody (Cat# 557355, BD biosciences) overnight at 4 °C and then washed with PBS. After isolation, mouse lung endothelial cells were cultured in endothelial cells medium supplemented with 10% FBS, 2% ECGS, 1% penicillin and streptomycin, and TGFβ inhibitor 1:1000 (Cat# SB431542, Medchemexpress).

### siRNA knockdown experiments

HUVECs and EA.hy926 were transfected using Lipofectamine 2000 (Cat# 11668027, Invitrogen) with duplex small interfering RNA (siRNA) against human NLRC5 (SASI_Hs02_00359503, Sigma, 50 nM) or human STAT3 (50 nM, GenePharma) and control siRNA (HSS 12935-300, Sigma, 50 nM/20 nM) for 48 h.

### *In vitro* adenovirus preparation and infection

HUVECs were seeded in 6-well-plates over 70% confluence and used for *in vitro* adenovirus infection. The NLRC5 and control adenovirus, which were constructed and purchased from Shanghai Genechem Co, Ltd, were added into the serum-free medium at the concentration of 50 mM for 24 h. The medium was replaced by the complete medium for another 24 h and used for further experiments.

### Construction and transfection of plasmid

STAT3-Flag, ΔNTD domain-Flag (2-120 aa delated), ΔCCD domain-Flag (141-313 aa delated), ΔDBD domain-Flag (325-464 aa delated), ΔSH2 domain-Flag (584-647 aa delated);myc-NLRC5 NACHT (222-539 aa), myc-NLRC5 DD+NACHT (1-539 aa), myc-NLRC5 DD (1-221 aa) were constructed and purchased from Shanghai Genechem Co, Ltd. The following plasmids were generated from pGV219 vector with myc tag including myc-NLRC5 full length (Cat# 37509, Addgene), myc-NLRC ΔDD (Cat# 37511, Addgene) and obtained from Addgene Institution.

### Immunofluorescence Staining and Fluorescence Confocal Microscopy

We injected 10 mL of PBS/Heparin (Cat# H3149, Sigma-Aldrich, 10 U/mL) and 10 mL of 4% paraformaldehyde solution consecutively at a rate of 2 mL/min into left ventricle of the mice heart. Gastrocnemius muscles were collected after perfusion fixation. Tissue samples were transferred into 30% sucrose solution at 4 °C overnight and embedded in optimal cutting temperature compound and frozen in liquid nitrogen, followed by sectioning at 8 mm thickness. Sections were incubated in primary antibodies (Table S1.1). DAPI 1:5000 (Cat# ab228549, Abcam) was used as a nuclear counterstain. Tissue was visualized using objective on Nikon Eclipse TE-2000U immunofluorescent microscope (Nikon, Japan). The area and percentage of staining was quantified using NIS-elements v3.0 (Nikon) and expressed as a percentage of the total surface area of the tissue section. Necrotic area in the gastrocnemius muscle was analyzed by hematoxylin and eosin staining. Necrotic cells displayed a glassy homogeneous appearance in the cytoplasm with increased neutrophils.

### RNA Isolation and RT-qPCR Analysis

Total RNA was isolated using Trizol (Cat# 15596026, Thermo Fisher) and extracted by chloroform and isopropanol. The yield and purity of the total RNA was assessed by Nanodrop 2000 (Thermo Scientific). The total mass of 1000 ng RNA was taken for subsequent reverse transcription using PrimerScript RT Reagent Kit (Cat# RR047A, Takara, Japan). Then, qPCR was performed by KAPA SYBR FAST kit (Cat# KM4101, KAPA Biosystem) and using the Roche LightCycler96 (Roche). Each sample was run in triplicate. Data was normalized by using GAPDH as the control. The expression of target genes was compared with fold-change between reference control and experimental groups. The primers were listed in the Table S2.

### Extraction of Cytoplasmic and Nuclear Proteins

HUVECs were prepared in 6 cm dishes for extraction of cytoplasmic and nuclear proteins. In short, after infection of adenovirus for 48 h, HUVECs were starved for 4 h and IL-6 (20 ng/mL, Cat# 200-06, PeproTech) was added into the medium for 0, 15, 30, 60 min. HUVECs were then digested by 0.025% trypsin with EDTA and suspended in completed medium. The cytoplasmic and nuclear proteins were extracted following the protocol of NE-PERTM Nuclear and Cytoplasmic Extraction Reagent (Cat# 7883, Invitrogen). HUVECs were also stimulated with VEGFA-165 (50 ng/mL, 12 h) or LPS (100 ng/mL, 24 h), and then were subjected to the same procedures mentioned above.

### Western Blot Analysis

Whole cell lysis of HUVECs were harvested for western blot using 1× cell lysis (Cat# 9803, Cell Signaling Technologies) with protease inhibitors (Cat# 04693159001, Roche Molecular Biochemicals, USA). Lysates were centrifuged at the speed of 12,000 g at 4 °C for 10 min. The supernatants were quantified using bicinchoninic acid kit (Cat# 20201ES76, Yeasen) and subjected to SDS-polyacrylamide gel electrophoresis and electrotransferred onto PVDF membranes. Primary antibodies were listed on Table S1.1. The PVDF membranes were washed in PBST and then incubated in PBST containing a 1:5,000 dilution of indicated secondary antibodies listed on Table S1.2. HRP was detected using the Super Signal chemiluminescence reagent substrate (Cat# 32134, Thermo Fish Scientific) and signal was visualized on the Biorad Chemdoc system. All experiments were performed at least three times.

### Co-immunoprecipitation

HUVECs or HEK293T cells were harvested in 1× cell lysis buffer (Cat# 9803, Cell Signaling Technologies) with protease inhibitors (Cat# 04693159001, Roche). After centrifugation, 1000 µg of cell lysate was incubated with the indicated primary antibodies (Table S1.1) at 4 °C overnight. The lysate immunoprecipitated with anti-Immunoglobulin G antibody served as negative control. The immune complexes were then purified by 20 µl of protein A/G agarose (Cat# sc-2003, Santa Cruz) at 4 °C for 5 h and washed by precooled cell lysis buffer. The immunoprecipitated protein were then used for Western Blot.

### Edu Assay

After starvation, VEGFA-165 was used to stimulate HUVECs for 12 h. Edu solution was incubated with the cells for 8 h in cell incubator, and subsequently fixed in 4% paraformaldehyde solution. The images of Edu-positive cells were taken by Leica DMI6000 microscopy. Three or four different fields of each section were taken for each group. The rate of proliferation was calculated as Edu-positive cells/4',6-diamidino-2-phenylindole (DAPI). All the reagents were included in Click-iT™ EdU Cell Proliferation Kit for Imaging, Alexa Fluor™ 594 dye (Cat# 10339, Invitrogen) or Alexa Fluor™ 488 dye (Cat# C10637, Invitrogen).

### TUNEL Assay

HUVECs were treated with si*NLRC5* for 48 h. Then phosphate buffered saline (PBS) containing 2% hydrogen peroxide was added into cells for 5 min and washed with PBS 3 times for 5 min. TdT enzyme buffer were added for 5 min. Next, TdT enzyme reaction liquid was added to the cells and incubated for 1 h at 37 °C. HUVECs were washed with PBS, and peroxidase labeled anti-digoxin antibody were added to the cells for 30 min at room temperature. The results were obtained by measuring the TUNEL positive cells divided by the number of DAPI-positive cells per scope. All the reagents were included in the *In situ* Cell Death Detection Kit (Cat# 12156792910, Roche). The results were observed by Leica DMI6000 microscopy.

### Tube Formation Assay

The Matrigel (Cat# 354230, Corning Matrigel) was thawed on ice overnight. Then the liquid Matrigel was injected into precooled 96-well-plate (50 µl/well) and plates were placed into the cell incubator. 100 µl with 20,000 HUVECs were seeded into the well and incubated for 8 h. Each group was taken 4 replications. The pictures were also taken by Leica DMI6000 microscopy. The total length and branch points was calculated to determining the ability of angiogenesis.

### Migration assay

HUVECs in 6-well-plate (pretreated) at 100% confluence were scratched by tip. VEGFA-165 was then added into the medium to enhance the migration of HUVECs. 4 pictures (n = 4) were captured by Leica DMI6000 microscopy in 0 h, 12 h, 24 h after scratching. The area of migration was calculated as the difference between the initial area (S0) and the area measured at each time (St). The migration rate was defined as the migrated area divided by the initial area: Migration rate = (S0 - St)/S0 * 100%.

### Luciferase assays

pGL4.45 [luc2p/ISRE/Hygro] vector (REF# E414, Promega) and pGL4.47 [luc2p/SIE/Hygro] vector (REF# E4041, Promega) in combination with various vectors for luciferase assays were carried out using FuGene reagent (Cat# E2311, Promega) according to the manufacturer's protocol. Briefly, transfections contained 100 ng renilla expression vector, and 1 µg of sis induce element (SIE) and interferon sensitive response element (ISRE) luciferase reporter plasmid or control empty vectors were conducted for 24 h and IL-6 (20 ng/mL) was added for another 18 h. For siRNA knockdown in HEK293T cells, NLRC5 was knocked down for 24 h and subjected to the transfection of vectors and stimulation. Cytosolic fractions were prepared at 24 h post-transfection using the Promega kit (Cat# E1910, Promega). Samples were analyzed with a luminometer and normalized to renilla activity to calculate the transfection efficiency.

### ChIP assay

HUVECs cultured in 10 cm dishes were transfected with vector or NLRC5 for 48 h (8 dishes per group). 1% formaldehyde was used to treat cells to cross-link the DNA and protein for 10 min at room temperature. Glycine was added to stop fixation. Next, cells were harvested with cold PBS. Cell nucleus was centrifugated at 3000 rpm for 5 min at 4 °C and resuspended in nuclei lysis buffer. Nuclear lysates were treated with 0.15ul Micrococcal Nuclease for 30 min at 37 °C. The lysate was then sonicated to shear DNA into fragments ranging 150~ 900 bp and equal amounts of chromatin were incubated overnight with anti-STAT3 and anti-Flag antibodies as listed in Table S1.1. 30 ul ChIP-grade protein G magnetic beads were used to absorb the chromatin. The DNA was purified by adsorption column. All of the regents were included in the SimpleChIP® Enzymatic Chromatin IP Kit (Cat# 9003, Cell Signaling Technologies). The Primers used for ChIP assay were listed in Table S2.

### RNA sequencing

Sample preparation, sequencing, and alignment: human umbilical vein endothelial cells were transfected with siRNA (si*NLRC5* or negative control, 50 nM) for 48 h. After starvation for 12 h, VEGFA-165 (50 ng/mL) was used to stimulate HUVECs for another 12 h. RNA sequencing was conducted with the help of Genergy Bio-technology (China). Briefly, RNA was harvested using Trizol reagent, Illumina TruSeq RNA Sample Prep Kit (Cat# FC-122-1001) was used with 1 µg of total RNA for the construction of sequencing libraries. RNA libraries were prepared for sequencing using standard Illumina protocols, and RNA sequencing was subsequently performed using the Illumina novaseq6000 platform. Finally, differentially expressed genes were screened using DESeq (version 1.22.1). Heatmaps of selected genes were created by using conditional formatting tool in Microsoft Excel with the highest and lowest expression for each gene displayed as red and blue (row min and row max). Raw and processed data were deposited in GEO (accession GSE159377).

### GSEA analysis

Gene Set Enrichment Analysis (GSEA, version 4.1.0, the broad institute of MIT and Harvard) was used to discuss whether a genetically defined genome is statistically significant between the two groups 32. We used R studio to construct RNK file according to each gene's log2FoldChange and -log10P.value compared between two groups based on our RNA-seq data. Then the profile was uploaded to the GSEA software. GSEA was performed with default algorithm as 1000 permutations, minimum term size of 15, and maximum term size of 500. HALLMARK was served as our annotated gene sets, collected from the Molecular Signature Database 3.0 33. Enriched gene sets were assigned based on normal *P*-value < 0.05 and FDR q-value < 0.25.

### Generation of NLRC5 global mice and EC specific KO mice

All experimental procedures involving animals were performed in accordance with the guidelines of the National Institutes of Health for the care and use of laboratory animals (NIH Publication, 8^th^ Edition, 2011) and approved by the Animal Care and Use Committees of Shanghai Tenth People's Hospital.

Global NLRC5-KO mice were obtained from Shanghai Biomodel Organism and bred as previously described 34. To generate the EC-specific NLRC5-KO mice, 5'loxP sites were inserted in the upstream of exons 1 where the promoter of EGE-LJL-121 was located. The 3'loxP was inserted into intron1 that was big and loxP element would not interfere mRNA splicing. To minimize the possibility of disruption of EGE-LJL-12 expression, two loxP sites were inserted into non-conserved regions. Southern blot and PCR were used by amplifying the region with the specific primers to identify the existence of loxP (details shown in Figure S5). Then, the NLRC5^flox/flox^ mice were crossed with Tie2Cre mice (purchased from Biocytogen) to obtain Tie2Cre-NLRC5^flox/flox^ mice. The study received approval by the Animal Care and Use Committees of Shanghai Tenth People's Hospital for animal welfare.

### Femoral artery ligation

Animals (8-10 weeks old) were anesthetized with 2% isoflurane. The left superficial femoral artery was ligated proximal to the deep femoral artery and proximal to the branching of the tibial arteries, leaving nerve and vein intact. The skin was sutured with 6-0 monofilament sutures. Then, mice were anesthetized by 2% isoflurane and were put on the platform to scan the blood flow of functional recovery (moorLDI Laser Doppler Perfusion Imager, England). The recovery rate of blood flow was measured as blood flow of ligated leg/sham leg. The score of necrosis in mice could be evaluated 28 days after ligation. Scores were calculated as following, 0 = none necrosis; 1 = 1-3 fingertips peeling; 2 = 4-5 fingertips peeling; 3 = necrosis of 1-3 toes; 4 = necrosis of 4-5 toes; 5 = necrosis of 1/3 soles; 6 = necrosis of 2/3 soles; 8 = necrosis of the full foot; 10 = necrosis of 1/3 legs; 12 = necrosis of 2/3 legs; 14 = necrosis of the whole leg.

### Bone marrow transplantation

NLRC5^flox/flox^ and Tie2Cre-NLRC5^flox/flox^ (male, 6-8 weeks) were used as recipients and WT mice were used as donors (indicated in Figure 4A). Recipient mice were irradiated with 9 Gy of radiation before injection. After sacrificing the donors, clean femur and tibia were collected in cold and sterilized PBS. Bone marrow was flushed from the cut bone as intact as possible using 1 mL syringe needle filled with RPMI 1640 medium. Then, cells were centrifuged and suspended with 1 mL RPMI 1640 after filtration of 0.45 µm strainer. Cells were transplanted to recipients (1×10^7^, 400 µl/recipient) through tail vein injection.

### Retinal angiogenesis assay

The P5 pups were sacrificed and eyes were fixed in 4% paraformaldehyde for 2 h and dissected, flatted, and permeabilized in 0.1% Triton X-100 in PBS for 30 min. 10% normal goat serum was used to block the retina at 4 °C overnight. They were incubated in biotin conjugated isolectin B4 (Bandeiraea simplicifolia, Cat# L-2140; Sigma-Aldrich) 20 µg/mL in 1% BSA at 4 °C overnight. The streptavidin-fluorescein isothiocyanate conjugate (Cat# SA10002, Invitrogen™) was added to the retina for 30 min in the second day. After being washed in PBS, the retinas were flat mounted.

### Tumor transplant

The B16F10 murine melanoma cell line was a gift from Prof. Ping Wang of Tongji University. In brief, cells were digested and suspended (1×10^7^ cells/mL, 100 µl/C57BL/6 mice) in cold PBS, and then injected subcutaneously into the abdominal area of 6 to 8-weeks-old male NLRC5^flox/flox^ mice and Tie2Cre-NLRC5^flox/flox^ mice. When tumors started to form, tumor growth was measured every two days by measuring the length and width and the tumor of volume (mm^3^) was calculated as 4π/3×(width/2)^2^×(length/2). Tumor was harvested (tumor size smaller than 1,000 mm^3^) and weighted at 14 days after injection. The vessel density was detected as the CD31-positive cells/scope (4 scopes per mice, 6 mice per group).

### En-face staining

According to Kyung Ae Ko et al. 35, the aorta was exposed and incised after the mice were sacrificed. The aorta was then fixed in 4% paraformaldehyde in PBS for 5 min. After removal of fat and connective tissues, the endothelium inside the vessel was exposed longitudinally and permeabilized by 0.1% Triton X-100 in PBS 30 min and blocked in 10% goat normal serum overnight. The antibodies (Table S1.1) diluted with 0.5% normal serum were incubated with vessels overnight. The specific secondary antibody (Table S1.2) and DAPI were used to incubate with the aorta for 30 min. Last, photos of aorta were taken by Nikon Eclipse TE-2000U microscope (Nikon) with HeNe laser and driven by EZ-C1 Viewer v3.5 software (Nikon).

### Acute lung injury model

Male 8-10 weeks C57BL/6 mice were used for the acute lung injury model. In short, the mice were intraperitoneal injected with LPS (10 mg/kg). 6 h later, the mice were sacrificed and perfused with 10 mL 4% paraformaldehyde in PBS. Then, the left lung was fixed in optimal cutting temperature compound.

### Flow cytometry analysis

As described before 36, anesthetized mice (8-10 weeks, male) were used to collect the leg skeletal muscles. The muscles without fat and connective tissue were cut into small pieces. Tissue was then suspended and digested in 5 mL HBSS containing 1 mL (100 U/mL) collagenase II (Cat# 1148089, Sigma-Aldrich) and 2 mL (1 U/mL) dispase (Cat# 354235, Corning) for 1 h at 37 °C. The mixture was filtered using sterile 0.45 µm nylon mesh and suspended in 1 U/mL dispase in HSBB. After digestion for another 30 min, the cells were filtered again. Centrifuged cells (1×10^6^, 100 µl) were blocked by 10% FBS for 30 min. The fluorescence labeled rat antibody (Table S1.2) was incubated with the cells in 1% FBS for 30 min. Finally, cells were fixed in 4% paraformaldehyde to perform flow cytometry analysis.

### Statistics

Student's *t*-test (two-sided), one-way ANOVA or two-way ANOVA followed by Bonferroni's pos-hoc test were used to calculate P value accordingly. Error bars represented standard errors, and numbers of experiments (n) were as indicated. *P* < 0.05 was regarded as significant.

## Results

### VEGFA-165 regulates the expression and redistribution of NLRC5 in ECs

**Figure 1:**
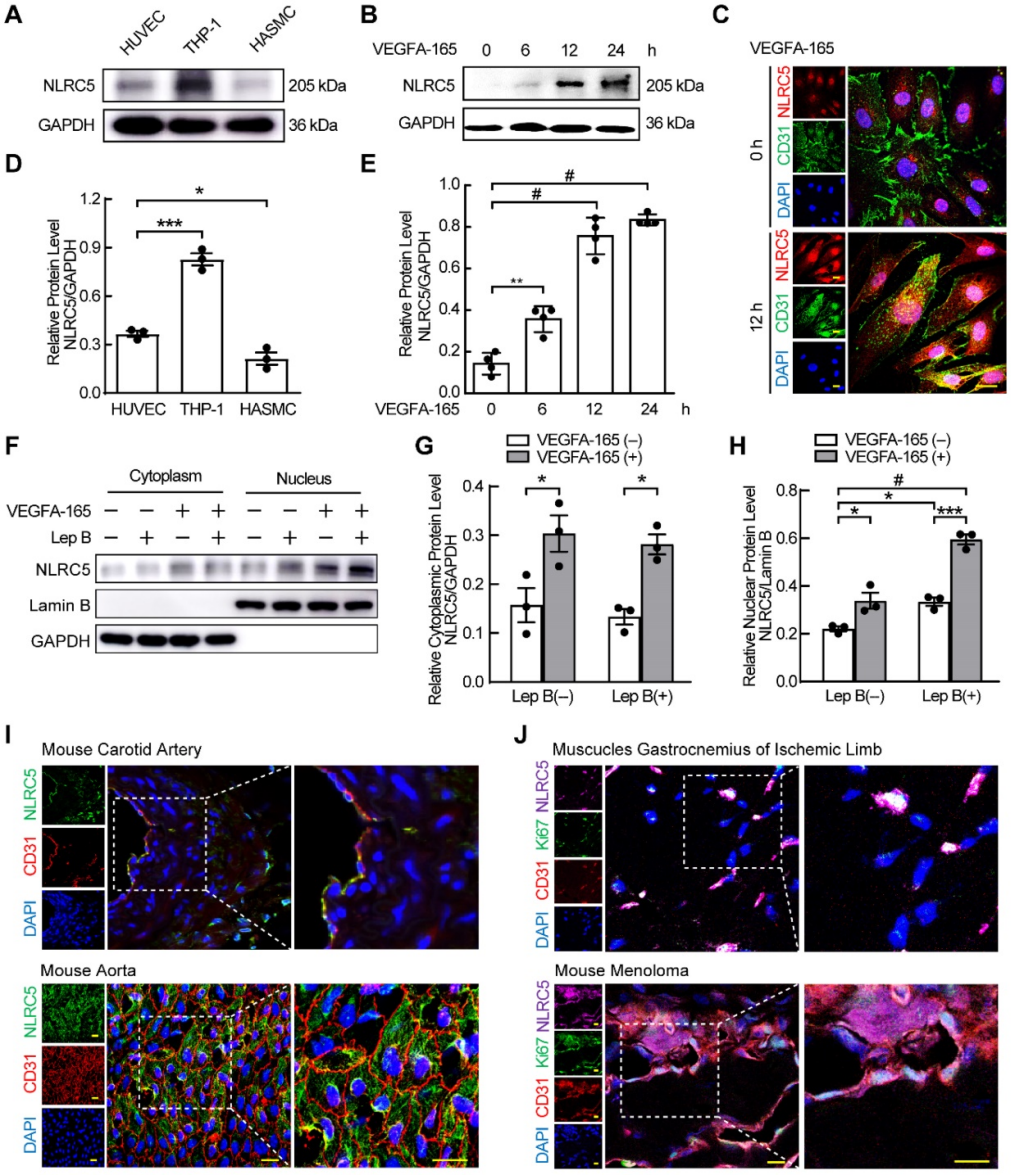
** NLR family CARD domain containing 5 (NLRC5) was increased by VEGFA-165 in human umbilical vein endothelial cells (HUVECs) and translocated into the nucleus.** (A) NLRC5 expression was moderately expressed in HUVECs compared to human myeloid leukemia mononuclear cells (THP-1) and human aortic smooth muscle cells (HASMCs). The protein level of NLRC5 was detected by western blot analysis. (B) HUVECs were stimulated with VEGFA-165 (50 ng/mL) for different time points. The protein level of NLRC5 was detected by western blot analysis. (C) HUVECs were treated with VEGFA-165 (50 ng/mL) for 0 and 12 h. Representative confocal microscopy images of immunofluorescence staining for NLRC5 (green), CD31 (red), 4'-6-diamidino-2 -phenylindole (DAPI, blue). Scar bar, 20 µm. (D) Quantification of A. Data are mean ± SEM, n = 3 independent experiments. One-way ANOVA with Bonferroni post-test, * *P* < 0.05, *** *P* < 0.005. (E) Quantification of B. Data are mean ± SEM, n = 4 independent experiments. One-way ANOVA with Bonferroni post-test, ** *P* < 0.01, # *P* < 0.001. (F) HUVECs were treated with VEGFA-165 (50 ng/mL) for 12 h with or without leptomycin B (100 µM) pretreated for 6 h. Nuclear and cytoplasmic fractions were extracted from HUVECs. The protein level of NLRC5 was detected by western blot analysis. (G) Quantification of NLRC5 expression in the cytoplasmic fraction. (H) Quantification of NLRC5 expression in the nuclear fraction. Data are mean ± SEM, n = 3 independent experiments. Two-way ANOVA with Bonferroni post-test, * *P* < 0.05, *** *P* < 0.005, # *P* < 0.001. (I) NLRC5 was localized in the cytoplasm of vascular endothelial cells in static mouse vessels. Representative confocal microscopy images of immunofluorescence staining for NLRC5 (green), CD31 (red), Ki67 (magenta), DAPI (blue). Scar bar, 50 µm. (J) NLRC5 was localized in the nucleus of proliferative vascular endothelial cells in ischemic legs and melanoma tumors of mice. Representative confocal microscopy images of immunofluorescence staining for NLRC5 (green), CD31 (red), Ki67 (magenta), DAPI (blue). Scar bar, 50 µm.

We previously found that NLRC5 was expressed in the endothelium of carotid arteries 21. To understand the relative expression of NLRC5 in the vasculature, we measured and compared NLRC5 protein expression in HUVECs, HASMCs, and THP-1 cells. NLRC5 was expressed higher in HUVECs compared with HASMCs (Figure 1A and D), and significantly increased in HUVECs after VEGFA-165 stimulation for 12 h (0.14 ± 0.03 folds vs 0.75 ± 0.04 folds, *P* < 0.001, Figure 1B and E). In contrast, CIITA, another member of the NLRs family with similar structure and function of NLRC5, could not be detected in the static aortic endothelium, pulmonary endothelium or endothelial cells in the ischemic limb of mice (Figure S1A-C). *In vitro*, CIITA was still almost undetectable in HUVECs with or without VEGFA-165 stimulation (Figure S1D-E). Notably, the increased NLRC5 expression in ECs was mainly located in the nucleus of HUVECs after treated with VEGFA-165 as shown by immunofluorescence (Figure 1C). Leptomycin B has been reported to inhibit the export of NLRC5 in the nucleus as NLRC5 is shuttled from cytoplasm to nucleus continually 37. After HUVECs were pretreated with leptomycin B, they were stimulated with VEGFA-165 for another 12 h followed by cell separation for nuclear and cytoplasmic protein fractions. NLRC5 expression was blocked in the nucleus. In addition, the expression of NLRC5 in the nucleus could also be enhanced after VEGFA-165 stimulation (Figure 1F-H). In contrast, *in vivo* experiments demonstrated that NLRC5 expression was located in the cytoplasm of quiescent endothelium of mice or human vessels (Figure 1I and Figure S2A). VEGFA is known to be highly concentrated in ischemic tissues or tumors with high demand of oxygen and nutrition 38. Indeed, NLRC5 expression was detected in the nucleus of some ECs in the vessels of both ischemic limbs and melanoma tumors in mice (Figure 1J). Finally, these NLRC5 nuclear positive ECs were in a proliferative state as they were also Ki67 positive, a marker of cell proliferation 39 (Figure 1J).

Since LPS could enhance the expression of NLRC5, we stimulated HUVECs with LPS to further investigate the expression and subcellular distribution of NLRC5. As expected, LPS slightly enhanced the expression of NLRC5 after 24 h (Figure S2C). And the increased NLRC5 was mainly located in cytoplasm (Figure S2E-F). Nonetheless, LPS-induced NLRC5 expression was dominantly located in the cytoplasm of ECs both *in vivo* using an acute lung injury model and *in vitro* after LPS stimulation of HUVECs (Figure S2B-D). Taken together, these results suggest that NLRC5 may serve as an important functional role in the nucleus of VEGFA-165 treated ECs.

### Deficiency of NLRC5 decreases angiogenesis *in vitro*

**Figure 2:**
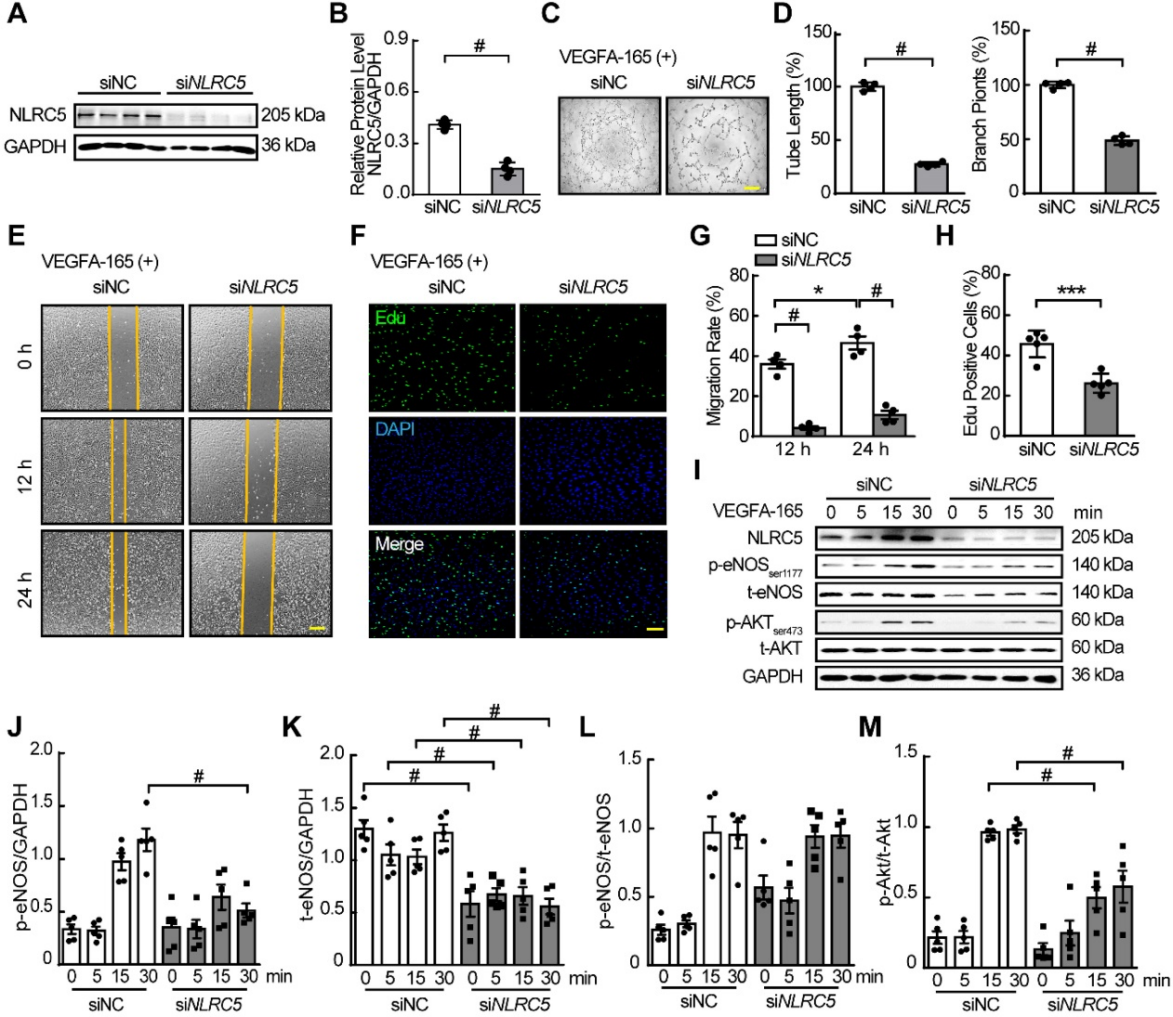
** siRNA knockdown of NLRC5 in HUVECs inhibited tube formation, migration, and proliferation and markedly decreased the phosphorylation of eNOS and AKT.** (A) siRNA knockdown decreased NLRC5 protein level in HUVECs. The expression of NLRC5 was detected by western blot analysis. (B) Quantification of A. Data are mean ± SEM, n = 4 independent experiments. Unpaired Student's *t*-test, # *P* < 0.001. (C) Tube formation of NLRC5 decreased HUVECs. Scar bar, 100 µm. (D) Quantification of total tube length and branch points. Data are mean ± SEM, n = 4 each group. Unpaired Student's *t*-test, # *P* < 0.001. (E) Migration of NLRC5 decreased HUVECs. Scar bar, 100 µm. (F) Proliferation of NLRC5 decreased HUVECs, 5-ethynyl-2'-deoxyuridine (Edu, green), DAPI (blue). Bar, 100 µm. (G) Quantification of C. Data are mean ± SEM, n = 4 each group. Two-way ANOVA with Bonferroni post-test, * *P* < 0.05, # *P* < 0.001. (H) Quantification of F. Data are mean ± SEM, n = 5 each group. Unpaired Student's *t*-test, *** *P* < 0.005. (I) p-AKT, p-eNOS decreased in NLRC5 knockdown HUVECs. (J-M), Quantification of I. Data are mean ± SEM, n = 5, independent experiments. Two-way ANOVA with Bonferroni post-test, # *P* < 0.001.

As VEGFA was a strong proangiogenic cytokine by promoting EC migration, survival, proliferation, and permeability in ischemic or neoplastic tissues 38, we hypothesized that the function of NLRC5 in the EC nucleus stimulated by VEGFA was to regulate angiogenesis. To prove this hypothesis, we first used siRNA to knockdown the expression of NLRC5 in HUVECs (siNC 0.41 ± 0.03 folds vs si*NLRC5* 0.15 ± 0.02 folds, *P* < 0.001) (Figure 2A-B). In response to NLRC5 knockdown, tube formation was significantly decreased as quantified by total tube length or the number of branch points (Figure 2C-D). After VEGFA stimulation, the si*NLRC5* knockdown groups exhibited lower migration rates at 12 and 24 h as quantified by scratching (Figure 2E and G), and proliferation by Edu staining (Figure 2F and H). On the other hand, TUNEL assay revealed that the decreased expression of NLRC5 had no significant influence on apoptosis (Figure S3). Moreover, the deficiency of NLRC5 in ECs led to decreased phosphorylation of Akt and phosphorylation of endothelial nitric oxide synthase (eNOS), two important signal pathways for angiogenesis 40 (Figure 2I-M).

The function of NLRC5 on endothelial cells was further investigated by primary mouse lung endothelial cells (MLECs) isolated from global NLRC5 KO and WT mice. Ad*NLRC5* was also used to rescue the expression of NLRC5 in MLECs isolated from KO mice (Figure S4A and B). The overexpressed NLRC5 would translocate into nucleus in VEGFA stimulated MLECs as well (Figure S4C). Certainly, MLECs isolated from KO mice exhibited decreased tube formation, which could be improved by the overexpression of NLRC5 (Figure S4D-F). Moreover, the decreased migration (Figure S4G-H) and proliferation (Figure S4I-J) of NLRC5 knockout MLECs were significantly rescued in the presence of NLRC5 overexpression. Collectively, these data indicate that NLRC5 expression is critical for EC angiogenic properties such as proliferation and migration, and suggest that it may play a role in pathological angiogenesis.

### Downregulation of NLRC5 inhibits pathological angiogenesis *in vivo*

To investigate the function of NLRC5 in angiogenesis *in vivo*, the NLRC5^flox/flox^ mice were crossed with Tie2Cre mice to generate Tie2Cre-NLRC5^flox/flox^ mice in which NLRC5 was knocked out specifically in ECs and myeloid/microglia 41 (Figure S5A-C). The deletion of NLRC5 was confirmed by qPCR in mouse lung tissues of the Tie2Cre-NLRC5^flox/flox^ mice (Figure S5D). Since angiogenesis took place in early embryo development, defined as physiological angiogenesis, we used the postnatal retina to investigate the role of NLRC5 in physiological angiogenesis. No significant changes of outgrowth length, superficial plexus, and sprout filopodia were observed in the retina of NLRC5^flox/flox^ mice compared with Tie2Cre-NLRC5^flox/flox^ mice (Figure S6A-F). Consistent with these findings, the expression of NLRC5 in the retina of mouse pups was fairly low in the early postnatal days, and increased after completion of angiogenesis in the superficial retina (Figure S6G-H). This might be the main reason for the negligible contribution of NLRC5 in retina angiogenesis.

**Figure 3:**
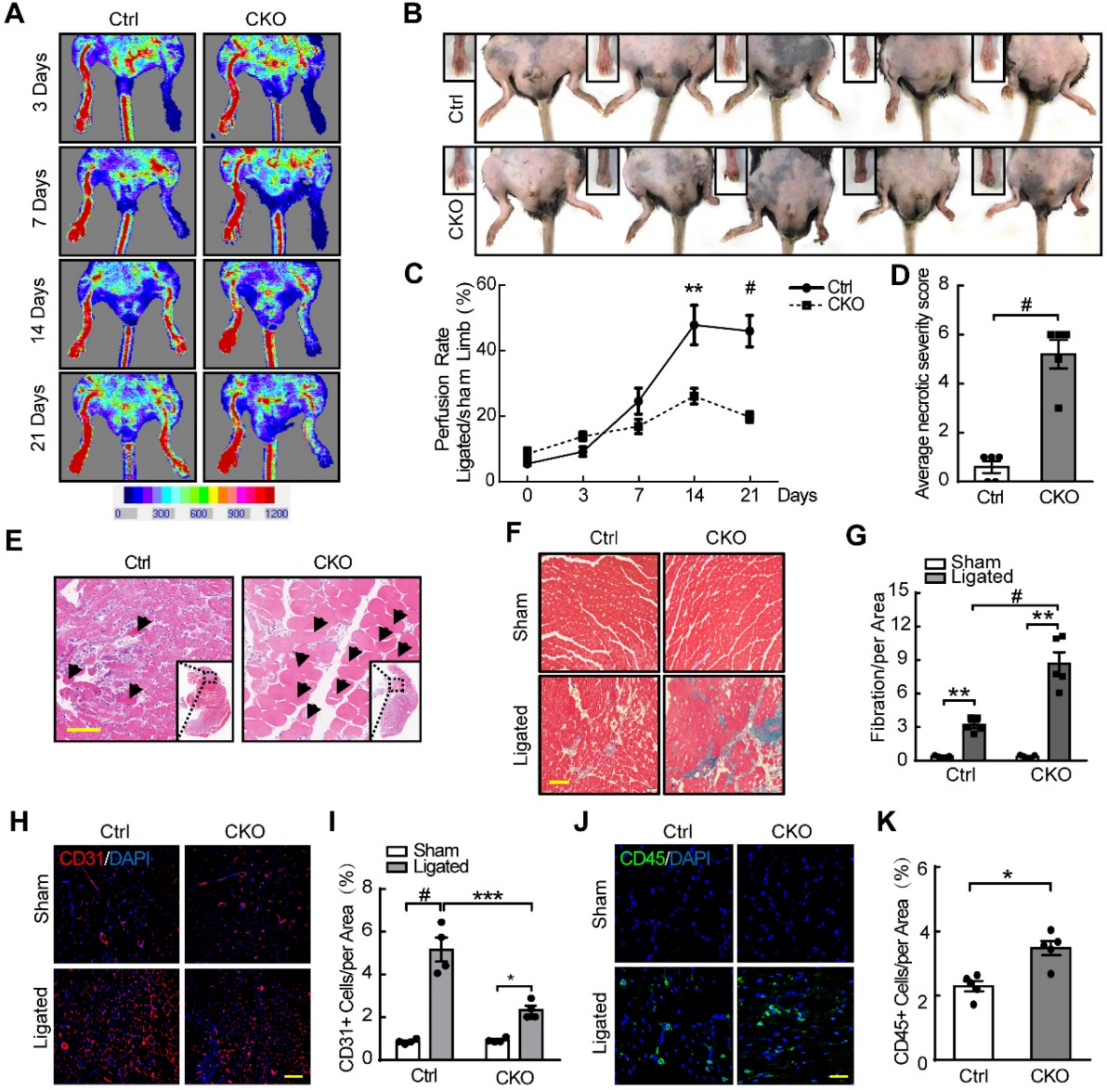
** Decreased angiogenesis of Tie2Cre-NLRC5^flox/flox^ mice resulted in severe necrosis and fibrosis of ischemic legs.** (A) The blood flow recovery in Tie2Cre-NLRC5^flox/flox^ (CKO) and NLRC5^flox/flox^ (Ctrl) mice. Blood flow was measured by tissue Doppler analysis. (B) The necrosis was quantified in Tie2Cre-NLRC5^flox/flox^ mice and NLRC5^flox/flox^ mice (28 days). (C) Quantification of A. n = 5 mice/group. Two-way ANOVA with Bonferroni post-test, ** *P* < 0.01, # *P* < 0.001. (D) Quantification of B. The average necrotic severity score. n = 5 mice/group. Unpaired Student's *t*-test, # *P* < 0.001. (E) Hematoxylin-eosin (HE) staining for necrotic cells in the cross section of ligated musculus gastrocnemius muscle (3 days). Scar bar, 50 µm. (F) Masson staining for collagen deposition in the cross section of musculus gastrocnemius muscle after femoral artery ligation (28 days). Scar bar, 100 µm. (G) Quantification of F. n = 5 mice /group, 4 scopes/mice. Data are mean ± SEM, two-way ANOVA with Bonferroni post-test, ** *P* < 0.01, # *P* < 0.001. (H) CD31-positive cells in the cross section of musculus gastrocnemius muscles after femoral artery ligation (14 days). Representative confocal microscopy images of immunofluorescence staining for CD31 (red), DAPI (blue). Scar bar, 100 µm. (I) Quantification of H. n = 4 mice/group, 3 scopes/mice. Two-way ANOVA with Bonferroni post-test, * *P* < 0.05, *** *P* < 0.005, # *P* < 0.001. (J) CD45-positive cells in the cross section of musculus gastrocnemius muscles after femoral artery ligation (14 days). Representative confocal microscopy images of immunofluorescence staining for CD45 (green), DAPI (blue). Scar bar, 100 µm. (K) Quantification of J. n = 5 mice/group, 3 scopes/mice. Two-way ANOVA with Bonferroni post-test, * *P* < 0.05.

We further investigated the role of NLRC5 in pathological angiogenesis using femoral artery ligation as a limb ischemia mouse model. The blood flow recovery in the ischemic limbs of Tie2Cre-NLRC5^flox/flox^ mice was significantly lower compared with control at day 14 (Tie2Cre-NLRC5^flox/flox^ 26.11 ± 2.38% vs NLRC5^flox/flox^ 47.86 ± 6.0%, *P* < 0.01) and after 21 days (Tie2Cre-NLRC5^flox/flox^ 19.75 ± 1.67% vs NLRC5^flox/flox^ 46.0 ± 0.7%, *P* < 0.001) (Figure 3A and C). More severe myocyte necrosis was observed on the early time point (3 days) after femoral artery ligation (Figure 3B, D-E), while a greater degree of fibrosis was observed later at 28 days (Tie2Cre-NLRC5^flox/flox^ 8.21 ± 2.11% vs NLRC5^flox/flox^ 2.08 ± 0.41%, *P* < 0.001) (Figure 3F-G). Using immunofluorescence, we found that CD31-positive cells in the ischemic area was significantly reduced in knockout mice compared with the controls (Figure 3H-I). Moreover, CD45-positive cells in the ischemic area were more increased in knockout mice (Figure 3J-K), supporting that inflammation was involved in angiogenesis post-ischemia. In contrast, the enlargement of arteries measured by artery diameter remained unchanged between groups (Figure S7A-B). These findings suggested that NLRC5 mainly influenced angiogenesis, but not arteriogenesis.

**Figure 4:**
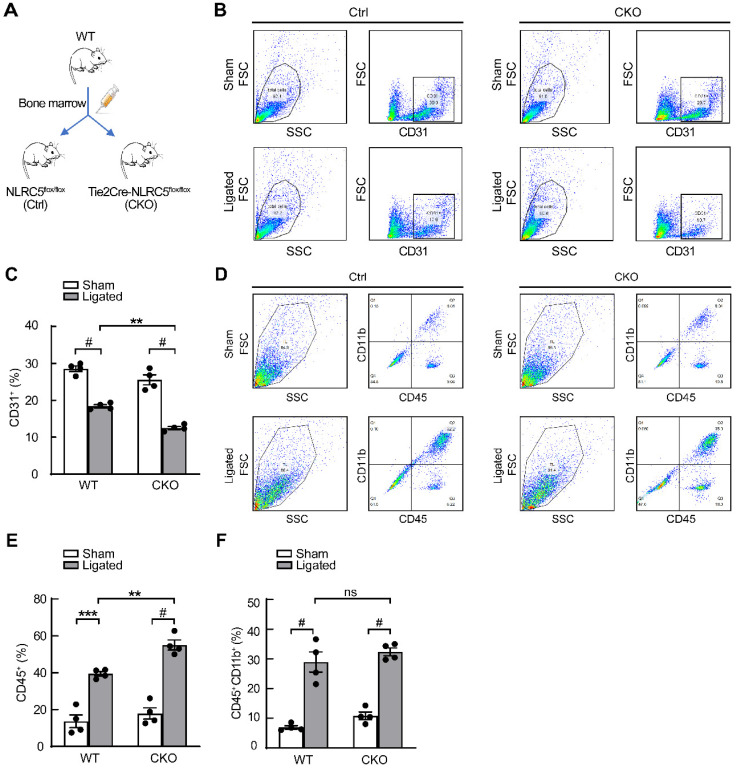
** Tie2Cre-NLRC5^flox/flox^ mice exhibited severe inflammation and decreased angiogenesis without the contribution of myeloid cells.** (A) Schematic of bone marrow transplantation studies. WT mice were the donors. NLRC5^flox/flox^ (Ctrl) mice and Tie2Cre-NLRC5^flox/flox^ (CKO) mice were the recipients. (B) The CD31^+^ cells were measured by fluorescence activated cell sorter (FACS). (C) Quantification of B. n = 4 mice/group. Data are mean ± SEM, two-way ANOVA with Bonferroni post-test, ** *P* < 0.01, # *P* < 0.001. (D) The CD45^+^CD11b^+^ cells were measured by fluorescence activated cell sorter (FACS). (E-F) Quantification of D. n = 4 mice/group. Data are mean ± SEM, two-way ANOVA with Bonferroni post-test, ** *P* < 0.01, *** *P* < 0.005, # *P* < 0.001.

As NLRC5 was also known to be expressed in myeloid cells 42, bone marrow transplantation studies were conducted to exclude the effect of myeloid cells-derived NLRC5 on angiogenesis (Figure 4A). The data from flow cytometry analyses demonstrated that Tie2Cre-NLRC5^flox/flox^ mice still had less CD31-positive cells compared with NLRC5^flox/flox^ mice after they were transplanted with WT bone marrow (Tie2Cre-NLRC5^flox/flox^ 12.5 ± 0.43% vs NLRC5^flox/flox^ 18.4 ± 0.42%, *P* < 0.01, Figure 4B-C). Despite the greater level of CD45^+^ inflammatory cells that accumulated in the local injured tissues there was no difference of bone marrow-derived CD45^+^CD11b^+^ myeloid cell recruitment in the injured legs between groups (Figure 4D-F). Taken together, these findings indicate that there is a limited role of bone marrow-derived cells, particularly myeloid cell-derived NLRC5-deficient cells, contributing to the decreased angiogenesis observed in Tie2Cre-NLRC5^flox/flox^ mice.

Finally, as angiogenesis was essential in tumor growth through which tumor tissues gain its nutrient supply. Melanoma is a cutaneous neoplasia with rapid angiogenesis, which might dramatically increase the risk of lethality 43. And the expression of NLRC5 was correlated with survival rate in patients with melanoma which suggested that NLRC5 might have effect on the procession of melanoma 44. As a result, we used melanoma as another model that mimics a complicated microenvironment and further explored the contributing role of NLRC5 in angiogenesis. The melanoma cells (B16F10) were injected into mice subcutaneously. Remarkably, tumors in Tie2Cre-NLRC5^flox/flox^ mice grew slower than the NLRC5^flox/flox^ mice as quantified by final tumor size (Tie2Cre-NLRC5^flox/flox^ 56.95 ± 23.27 mm^3^ vs NLRC5^flox/flox^ 525.5 ± 112.8 mm^3^, *P* < 0.001) and tumor weight (Tie2Cre-NLRC5^flox/flox^ 19.5 ± 7.6 mg vs NLRC5^flox/flox^ mice, 249.5 ± 24.0 mg, *P* < 0.001) without much difference in body weight (Figure S8A-D). In addition, Tie2Cre-NLRC5^flox/flox^ mice had less vessel density (CD31+ cells number) (Figure S8E-F).

### NLRC5 binds with STAT3 but not STAT1 in VEGFA treated ECs

**Figure 5:**
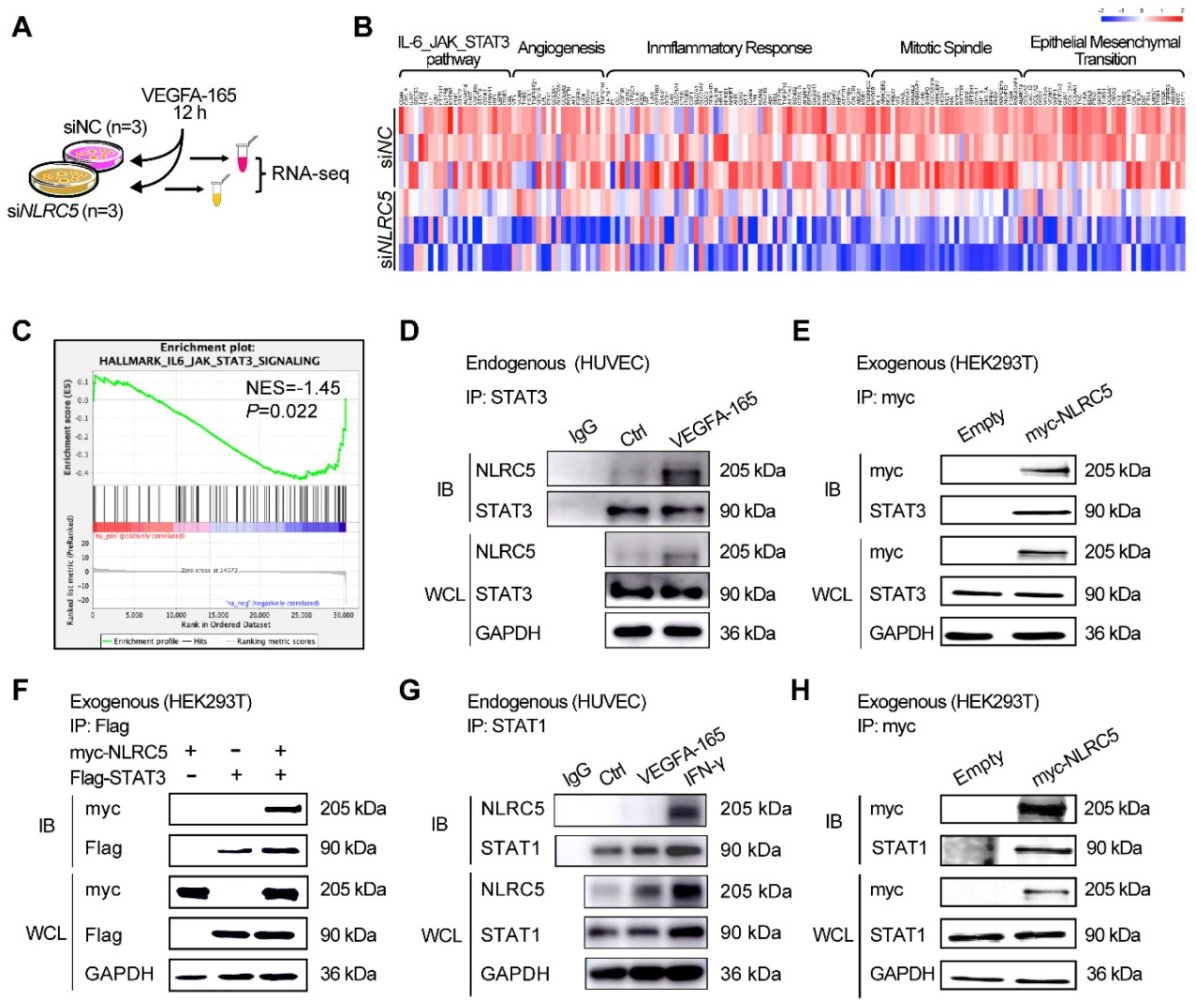
** Signal transducer and activator of transcription 3 (STAT3) bound with NLRC5 in VEGFA-165 stimulated ECs.** (A) Flow chart of sample preparation for RNA sequencing. (B) Heat maps of related genes based on RNA-seq. (C) Gene set enrichment analysis (GSEA) was performed using the gene in the IL-6-JAK-STAT3 pathway generated from RNA-seq. (D) Identification of STAT3 as a binding partner of NLRC5 in VEGFA-165 treated HUVECs. HUVECs were stimulated with VEGFA-165 for 0 or 12 h. The lysate was immunoprecipitated with anti-STAT3 antibody and then immunoblotted with the indicated antibodies. (E) Co-immunoprecipitation of myc-NLRC5 overexpressed HEK293T cells. The lysate was immunoprecipitated with anti-myc antibody and then immunoblotted with the indicated antibodies. The protein expression was detected by western blot analysis. (F) Co-immunoprecipitation of myc-NLRC5 in combination with Flag-STAT3 in HEK293T cells. The lysate was immunoprecipitated with anti-Flag antibody and then immunoblotted with the indicated antibodies. The protein expression was detected by western blot analysis. (G) Identification of STAT1 as a binding partner of NLRC5 in IFN-γ-treated HUVECs, but not in VEGFA-165-treated HUVECs. HUVECs were stimulated with IFN-γ and VEGFA-165 for 12 h. The lysate was immunoprecipitated with anti-STAT1 antibody and then immunoblotted with the indicated antibodies. (H) Co-immunoprecipitation of myc-NLRC5 overexpressed HEK293T cells. The lysate was immunoprecipitated with anti-myc antibody and then immunoblotted with the indicated antibodies. The protein level was detected by western blot analysis.

To investigate the potential mechanism of pro-angiogenesis effects of NLRC5, we conducted GSEA analysis based on our RNA-seq results. Several angiogenesis related pathways were found to be suppressed in the NLRC5 knock down group, the mainly enriched pathway and related genes' profile of GSEA results (Figure 5A-B). Of note, down regulation of NLRC5 was positively related to IL-6-JAK-STAT3 signaling pathway's suppression according to the GSEA analysis results (Figure 5C). This analysis gives us a clue to further explore the possible rationale for the interaction of NLRC5 and STAT3.

Based on previous studies, NLRC5 regulates gene expression through forming interactions with several transcription factors 21, 45. Our presented data indicated that NLRC5 might serve as a transcriptional regulator or coactivator in the nucleus of ECs. STAT3 is a transcription factor that has been clarified its function in angiogenesis beyond classical role in regulating tumorigenesis or inflammation 46. Lysates of HUVECs treated with VEGFA-165 were immunoprecipitated with anti-STAT3 antibodies. As shown in Figure 5D, the binding of STAT3 to NLRC5 was enhanced after VEGFA-165 stimulation. This finding was in line with the results of co-immunoprecipitation studies of NLRC5-myc and STAT3-Flag overexpressed in HEK293T cells (Figure 5E-F). Together, these results showed that STAT3 was enhanced in VEGFA-165 stimulated HUVECs and banded with NLRC5.

Because STAT1 and STAT3 shared more than 75% of their homology sequence 47, so it urged us to suppose that NLRC5 would also combine with STAT1. The results showed that STAT1 was a binding partner of NLRC5 in IFN-γ-treated HUVECs, but not in VEGFA-165 treated HUVECs (Figure 5G). And the result from co-immunoprecipitation of myc-NLRC5 overexpressed HEK293T cells revealed the binding of Flag-STAT1 and myc-NLRC5 as well (Figure 5H).

### DD+NACHT domain is a key domain for NLRC5 binding to STAT3 in the nucleus

**Figure 6:**
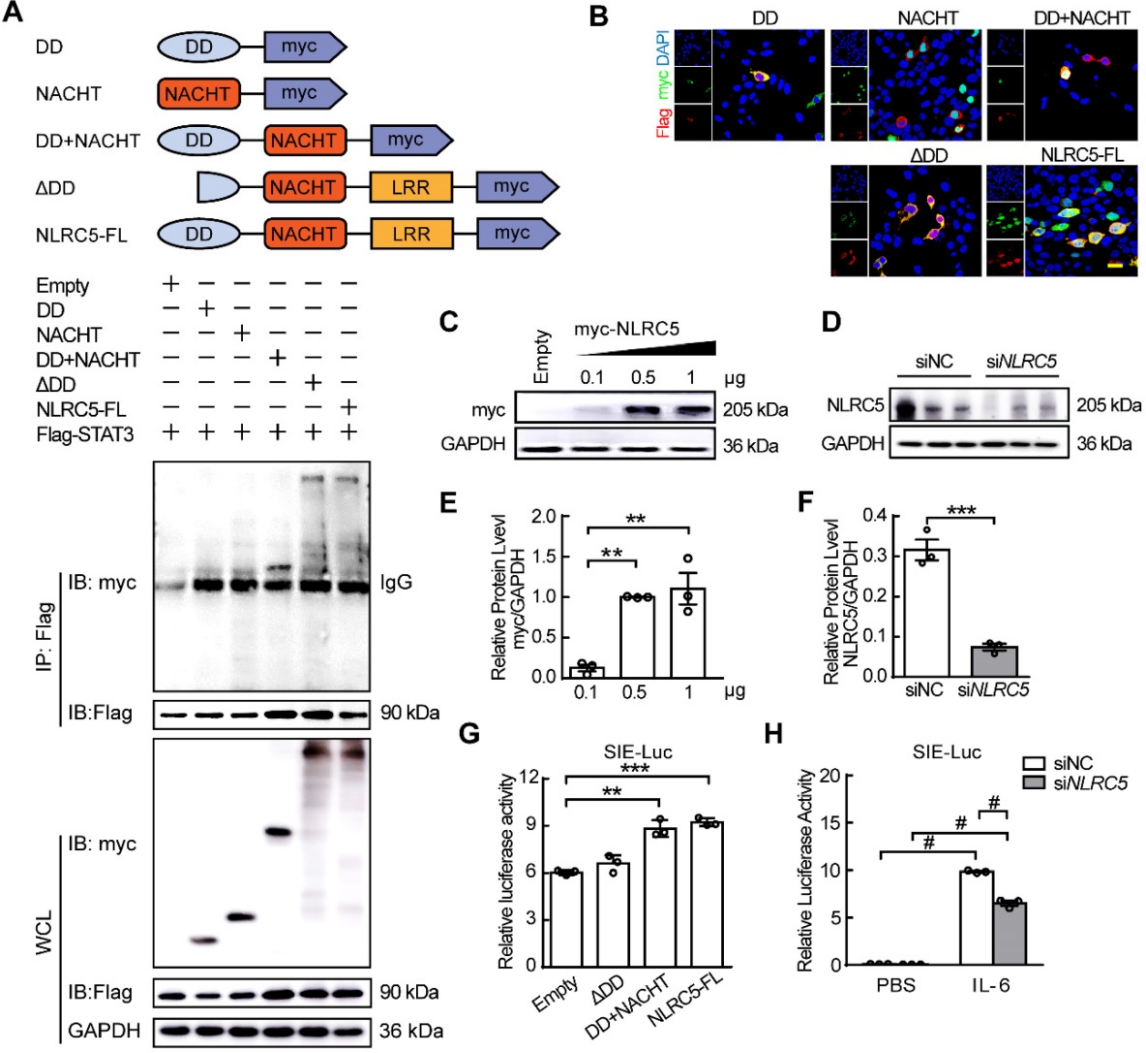
** The integrity of the DD+NACHT domain of NLRC5 was critical for NLRC5 transcriptional activity.** (A) Flag-STAT3 was co-transfected with myc-DD, myc-NACHT, myc-DD+NACHT, myc-ΔDD, and myc-NLRC5 full length plasmids, respectively, in HEK293T cells. The lysates were immunoprecipitated and then immunoblotted with antibodies against the indicated proteins. (B) Representative confocal microscopy images of immunofluorescence staining for myc (green), Flag (red), DAPI (blue). Scar bar, 20 µm. (C) myc-NLRC5 was transfected in HEK293T cells and the efficiency was detected by western blot analysis. (D) Endogenous NLRC5 was knockdown by siRNA. (E) Quantification of C. Data are mean ± SEM, n = 3 independent experiments. One-way ANOVA with Bonferroni post-test, ** *P* < 0.01. (F) Quantification of D. Data are mean ± SEM, n = 3 independent experiments. Unpaired Student's *t*-test, *** *P* < 0.005. (G) Sis induce element (SIE) promoter luciferase reporter plasmid was respectively transfected with myc-NLRC5, myc-DD, or myc-ΔDD into HEK293T cells for 24 h. Cells were treated with PBS or IL-6 (20 ng/mL) for another 18 h. Promoter activities were normalized to renilla luciferase. The results were expressed as relative luciferase activity. Data are mean ± SEM, n = 3 independent experiments. One-way ANOVA with Bonferroni post-test, ** *P* < 0.01, *** *P* < 0.005. (H) After transfection, the cells were stimulated with IL-6 (20 ng/mL) for 18 h. Promoter activities were normalized to renilla luciferase. Data are mean ± SEM, n = 3 independent experiments. Two-way ANOVA with Bonferroni post-test, # *P* < 0.001.

NLRC5 is an atypical member of the NLRs family because of its unusual caspase activation and recruitment domain (CARD domain or DD domain) and its longest leucine rich repeats (LRRs domain) 7. As our data suggested above, NLRC5 interacted with STAT3 in ECs. To investigate which specific domain of NLRC5 mediated this interaction, several plasmids containing different NLRC5 domains (myc-tagged) including the DD domain (1-221aa), NACHT domain (222-539 aa), DD+NACHT domain (1-539 aa), ΔDD domain (135-1855 aa) and the NLRC5 full-length plasmid were transfected together with STAT3-Flag in HEK293T cells. Co-immunoprecipitation showed that while neither DD nor NACHT domains interacted with STAT3, the DD+NACHT domain did bind STAT3 (Figure 6A). Moreover, the mutant ΔDD domain, 1-134 amino acids deleted from the N-terminal of NLRC5, also bound to STAT3 as well (Figure 6A). Yet, this ΔDD domain only localized to the cytoplasm while DD+NACHT domain as well as the NLRC5 full-length plasmid could shuttle into nucleus besides interacting with STAT3 (Figure 6B). Further, domain deleted STAT3 plasmids were transfected into HEK293T in combination with NLRC5 respectively. Co-IP was conducted to determine which main domain of STAT3 (Flag-tagged) interacted with NLRC5 (myc-tagged). As shown in Figure S9A, ΔNTD domain (2-120 aa deleted), ΔCCD domain (141-313 aa deleted), ΔDBD domain (325-464 aa deleted) and ΔSH2 domain (584-647 aa deleted) were constructed. And the result of Co-IP also has clarified CCD domain was the main domain for the interaction of NLRC5 and STAT3 (Figure S9B). In brief, these results indicated that the structural integrity of DD+NACHT domain was essential for the interaction of NLRC5 and STAT3 in the nucleus.

To explore the effects of the various domains on the expression of the downstream STAT-responsive elements, the SIE luciferase reporter was transfected along with the DD+NACHT domain, ΔDD domain, or the full length NLRC5 into HEK293T cells and stimulated with IL-6. Although the DD+NACHT domain and full length NLRC5 could enhance the relative luciferase activity, the ΔDD domain remained unchanged (Figure 6C, E and G). In addition, knockdown of NLRC5 in HEK293T cells inhibited the relative luciferase activity significantly (Figure 6D, F and H). Taken together, these data indicated that the DD+NACHT domain is necessary for the ability of NLRC5 to maintain its transcriptional activity.

### NLRC5 promotes STAT3 transcriptional activity by enhancing the accumulation of unphosphorylated STAT3 in the nucleus

**Figure 7:**
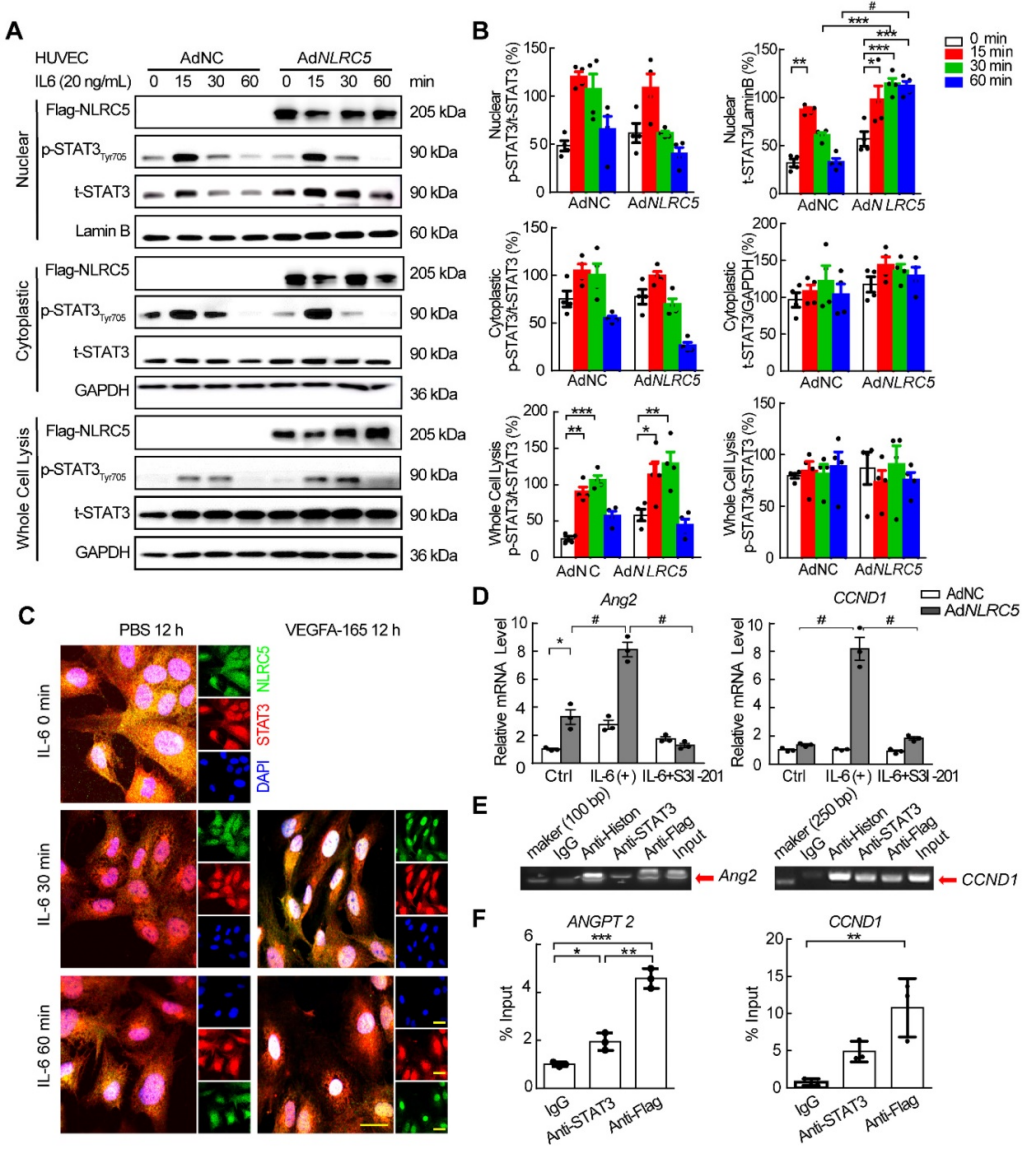
** Overexpressed NLRC5 prolonged the accumulation of STAT3 in the nucleus after interleukin 6 (IL-6) stimulation in HUVECs.** (A) HUVECs were transfected with AdNC or Ad*NLRC5* for 48 h and then treated with IL-6 (20 ng/mL) for the indicated time points over 60 min. Nuclear and cytoplasmic fractions were extracted from HUVECs. The protein expression of Flag-NLRC5, t-STAT3, p-STAT3(Tyr705), Lamin B, and GAPDH were measured by western blot analysis. (B) Quantification of A. Data are mean ± SEM. n = 4 independent experiments, two-way ANOVA with Bonferroni post-test, * *P* < 0.05, ** *P* < 0.01, *** *P* < 0.005, # *P* < 0.001. (C) Representative confocal microscopy images of immunofluorescence staining for Flag-NLRC5 (green), STAT3 (red) and DAPI (blue). Scar bar, 20 µm. (D) NLRC5 overexpression increased mRNA expression of angiopoietin-2 and cyclin D1 in IL-6 induced HUVECs, and the enhancement was inhibited by the STAT3 specific inhibitor S3I-201. Data are mean ± SEM, n = 3 independent experiments. Two-way ANOVA with Bonferroni post-test, * *P* < 0.05, # *P* < 0.001. (E-F) ChIP assay for the promoter of angiopoietin-2 and cyclin D1. HUVECs were transfected with Ad*NLRC5* and the sonicated nuclear lysates were incubated with anti-STAT3 or anti-Flag antibodies. The purified DNA was amplificated by qPCR and confirmed by southern blot. Data are mean ± SEM, n = 3 independent experiments. Two-way ANOVA with Bonferroni post-test, * *P* < 0.05, ** *P* < 0.01, *** *P* < 0.005.

As it had been reported that the unphosphorylated form of STAT3 accumulated in the nucleus to regulate gene expression 48, 49, we further hypothesized that NLRC5 might regulate the STAT3 function by binding and trapping unphosphorylated STAT3 in the nucleus. To verify this hypothesis, NLRC5-Flag tagged adenovirus was constructed and used to infect HUVECs stimulated with interleukin-6 (IL-6). The overexpressed NLRC5 delayed the translocation of unphosphorylated STAT3 out of the nucleus by 30 min after IL-6 stimulation in HUVECs and was sustained until 60 min (AdNC 32.63 ± 4.18 folds vs Ad*NLRC5* 114.74 ± 5.19 folds, *P* < 0.001) (Figure 7A-B). Similar results were further confirmed by immunofluorescence staining (Figure 7C). Overexpression of NLRC5 in HUVECs also promoted some STAT3-regulated genes expression such as angiopoietin-2 (Ang2) and cyclin D1 (CCND1) (Figure 7D and Figure S10), suggesting the subsequent transcriptional activity brought about by the trapped unphosphorylated STAT3. Furthermore, such enhanced transcriptional activity could be blocked by S3I-201, an inhibitor of STAT3 50 (Figure 7D). ChIP assay of the Ang2 and CCND1 promoter provided further evidence that the binding of NLRC5 and STAT3 in the nucleus increased the expression of Ang2 or cyclin D1 targeted to the promoter region (Figure 7E-F).

### Inhibiting STAT3 suppresses the proangiogenic properties of NLRC5 in ECs

**Figure 8:**
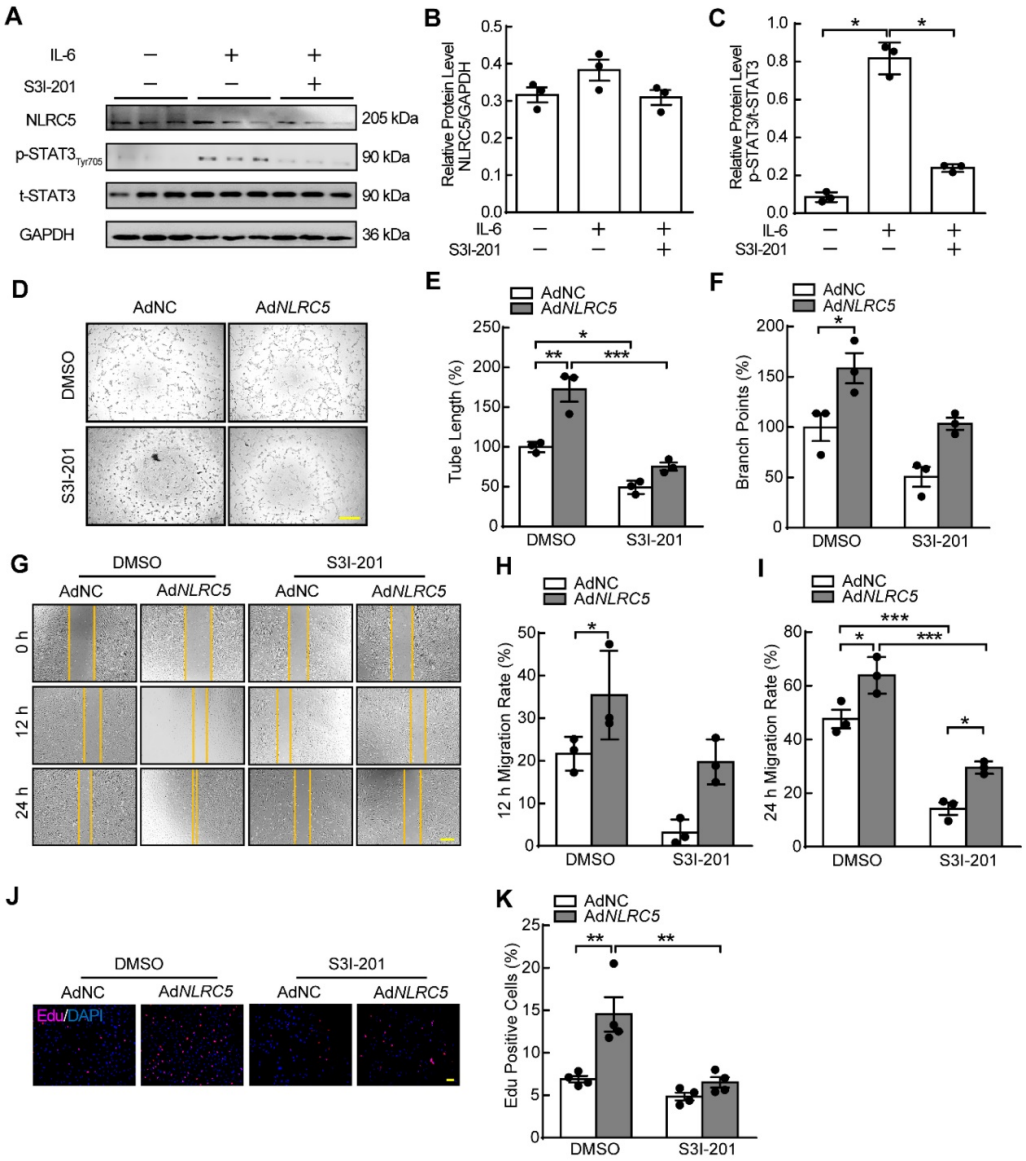
** STAT3 specific inhibitor S3I-201 impaired the induced enhancement of EC tube formation, migration, and proliferation mediated by NLRC5 overexpression.** (A) The efficiency of S3I-201 to bind with STAT3 and to decrease the phosphorylation of STAT3. (B-C) Quantification of A. Data are mean ± SEM, n = 3 independent experiments. Two-way ANOVA with Bonferroni post-test, * *P* < 0.05. (D) S3I-201 inhibited the tube formation in NLRC5 overexpressed in HUVECs. Scar bar, 100 µm. (E-F) Quantification of D. Data are mean ± SEM, n = 3 independent experiments. Two-way ANOVA with Bonferroni post-test, * *P* < 0.05, ** *P* < 0.01, *** *P* < 0.005. (G) S3I-201 inhibited the migration in NLRC5 overexpressed in HUVECs. Scar bar, 100 µm. (H-I) Quantification of G. Data are mean ± SEM, n = 3 independent experiments. Two-way ANOVA with Bonferroni post-test, * *P* < 0.05, *** *P* < 0.005. (J) S3I-201 inhibited the proliferation in NLRC5 overexpressed in HUVECs. 5-ethynyl-2'-deoxyuridine (Edu, red), DAPI (blue). Scar bar, 100 µm. (K) Quantification of J. Data are mean ± SEM, n = 4 independent experiments. Two-way ANOVA with Bonferroni post-test, ** *P* < 0.01.

STAT3 inhibitor S3I-201 and siRNA were used to block the activity of STAT3. The phosphorylation of STAT3 induced by IL-6 stimulation was significantly inhibited without altering expression of NLRC5 when HUVECs were treated with S3I-201 (Figure 8A-C). At the same time, the protein level of STAT3 was suppressed after si*STAT3* knockdown in HUVECs. S3I-201and si*STAT3* reversed the proangiogenic properties of overexpressed NLRC5 on EC tube formation (Figure 8D-F & Figure S11A-C), migration (Figure 8G-I & Figure S11D-E), and proliferation (Figure 8J-K & Figure S11F-G). Moreover, overexpression of STAT3 (WT) rather than STAT3 (Y705F) (Figure S12A-C) has rescued the decreased angiogenesis in NLRC5 defected endothelial cell line. Taken together, inhibition of STAT3 activity significantly reduced NLRC5-mediated pro-angiogenic properties in ECs.

## Discussion

In this study, we demonstrate the existence of an intertwined regulation between the VEGF and IL-6/STAT3 signaling pathways and pathological angiogenesis. NLRC5 promotes pathological angiogenesis through its interaction with STAT3 and subsequent upregulation of angiogenesis-related genes.

Prior studies of NLRC5 mainly focused on its role in the inflammatory response as it was first reported in the cytoplasm of hemocytes 21. Consistent with these studies, we found that LPS could slightly enhance the cytoplasmic expression of NLRC5 in ECs *in vitro* or in MLECs from an acute lung injury model induced by LPS *in vivo*. Such effect was attributed to cytoplasmic NLRC5 as the LPS did not change the subcellular distribution of NLRC5 protein. NLRC5 could also regulate the expression of ICAM-1 and VCAM-1 in ECs (Figure S2G), raising the possibility that NLRC5 in the cytoplasm might suppress NF-κB activities and decrease the expression of ICAM-1 and VCAM-1.

Data from our experiments further suggested that NLRC5 in ECs served as a transactivator of STAT3 and stimulated angiogenic gene expression. Induction of angiogenesis by NLRC5 stemmed largely by its interaction with STAT3 since we did not find the interaction of STAT1. Phosphorylated STAT1 homodimers induced by IFN-γ are known to enhance ISRE, which eventually increased NLRC5 expression 51, 52. Although STAT1 could bind to NLRC5 following IFN-γ stimulation in HUVECs, STAT1 did not interact with NLRC5 in VEGFA-165-stimulated HUVECs. In line with these data, our results also demonstrated that STAT1 bound with NLRC5 in HEK293T cells and enhanced ISRE luciferase activities (Figure S13), meaning that the feedback mechanism of NLRC5 and STAT1 was more intricate.

STAT1 and STAT3 share more than 75% of their homology sequence 47 and both can recognize similar DNA consensus sequences based on a TTCNNN(T, G)AA motif 53. However, STAT1 often has the opposite function to STAT3 in a range of cellular paradigms such as inflammation or angiogenesis 47, 54. Accumulating studies demonstrate that STAT3 regulates angiogenesis by increasing the proliferation or migration of ECs 55-57. Traditionally, the phosphorylated STAT3 dimer evoked by IL-6 or other factors will translocate to the nucleus and transactivate target genes 58-60. In fact, STAT3 could activates transcription in the form of unphosphorylated STAT3 as well as phosphorylated STAT3. A recent study has demonstrated that unphosphorylated STAT3 in the nucleus interacted with yes-associated protein and enhances angiogenesis of ECs 61. In addition to this, STAT3 could regulate transcription of related genes by acetylation, methylation and even palmitoylation^62^-^64^. It is hard to exclude from the interaction with other forms of STAT3 in the present experiments since they could be concluded as unphosphorylated STAT3. Meanwhile, our results showed that NLRC5 preferentially bound to STAT3 rather than STAT1 in VEGFA-165-treated ECs *in vitro*. Although IL-6 is generally thought to be a pro-angiogenic cytokine 65, 66, its role in angiogenesis is not fully elucidated. Some studies indicate that the pro-angiogenic ability of IL-6 depends on the presence of VEGF *in vivo* 67-69. Our study provides a clue that NLRC5 is not only an essential sensor for inflammation since NLRC5 expression increases in the complicated microenvironment of tissue injury, but also a signal transducer of injury to promote tissue repair, through neovascularization by coordinating with the VEGF and IL-6/STAT3 pathways. In short, the functional variability of NLRC5 in multiple microenvironments is likely attributed to its interactions with various transcription factors in response to specific ligands in different cell-types. The proangiogenic characteristics of NLRC5 may be attributed to its unique structure to form a loop to integrate STAT3. The other NLRs that lack the α-helix-riched caspase domain may have more difficult to form a loop. Hence, the DD+NACHT domain of NLRC5 could be a druggable target for clinical therapy of ischemic disease states.

Interestingly, tumor growth and angiogenesis were also markedly reduced in the melanoma tumor transplant model of Tie2Ce-NLRC5^flox/flox^ mice. Since NLRC5 regulates MHC class I antigen presentation and T cell responses, the deficient mice are potentially more susceptible to pathogen infection 66, 70. In our hand, we observed that some MHCI related genes such as HLA-B, HLA-F and HLA-G were suppressed in si*NLRC5* knockdown HUVECs (Figure S14). In theory, the NLRC5-deficient mice could also aggravate tumor progression as NLRC5/CITA plays a crucial role in human cancer immunity through the recruitment and activation of tumor killing CD8+ T cells 71. However, the strong inhibition of tumor growth that we observed suggests that the STAT3 function *in vivo* is largely depended on NLRC5. These results provide evidence that the multifunctional role of NLRC5 in the nucleus and the specific binding partner of NLRC5 and STAT3, determined the fate of cells.

There are several limitations in this study. First of all, we used Tie2Cre mice to generate knockout mice. Since Tie2 is expressed in both ECs and myeloid cells, the phenotype observed in Tie2Cre mice is not exclusively of ECs origin. To eliminate the effect of myeloid cells where NLRC5 is also expressed, we perform bone marrow transplantation. The data of cytometry analyses have indicated there was no significant difference of CD45^+^CD11b^+^ (myeloid cell) between ligated legs of CKO and Ctrl mice. The data suggest the role of NLRC5 from ECs rather than hematopoietic cell-origin myeloid cells on angiogenesis in hindlimb ischemia studies. Meanwhile, the increase of CD45^+^ cells suggested the enhanced accumulation of CD45^+^CD11b^-^ cells in CKO ligated limbs. Therefore, it is needed for us to explore the subset of these cells and their contribution to angiogenesis in further study. Secondly, HUVECs were used to study the mechanism of NLRC5 in angiogenesis, but not the primary neonatal endothelial cells. HUVECs are isolated from umbilical vein of neonate with great potential of proliferation and are regarded as an easy tool to investigate endothelium *in vitro* 72, 73. ECs isolated from neonatal KO and WT mice might have better performance in comparing their potential on angiogenesis. But they are easily to differentiate into fibroblast-like cells (mesenchymal cells) as reported 74, 75. Hence, they are not stable to be used to investigate the mechanism of NLRC5 on angiogenesis. Thirdly, we cannot exclude a participatory role for other NLRC5 interacting factors in the nucleus beyond STAT3, although our data strongly suggests that STAT3 is at least sufficient to mediate NLRC5's angiogenic properties *in vitro*. Finally, we cannot rule out various combinations of domains of NLRC5 mediating the STAT3 interaction in specific cells types or in response to divergent pro-angiogenic stimuli. Future studies will be of interest to clarify these points further.

In summary, we presented new insights for NLRC5 in pathological angiogenesis in this study. Endothelial NLRC5 deficiency *in vitro* or *in vivo* inhibited pathological angiogenesis, but had no effect on physiological angiogenesis. The accumulation of NLRC5 sustained the expression of unphosphorylated STAT3 in the nucleus, which in turn increased the STAT3-regulated gene Ang2 and proangiogenic properties. Finally, we identified that the DD+NACHT domain of NLRC5 was required for binding to STAT3. Collectively, these findings reveal an unanticipated role for NLRC5 as a key molecular switch in response to tissue injury to facilitate neovascularization during tissue regeneration.

## Supplementary Material

Supplementary figures and tables.
