# Supplementary material for: The subcellular redistribution of NLRC5 promotes angiogenesis via interacting with STAT3 in endothelial cells
Source: Theranostics. 2021 Mar 4;11(9):4483–501. doi: 10.7150/thno.54473 (PMC7977449; doi:10.7150/thno.54473)
Supplement: Supplementary file 1 — Supplementary figures and tables. [file thnov11p4483s1.pdf]

Figure S1. Major histocompatibility class II (MHC II) transactivator (CIITA) was minimally expressed in ECs. (A) CIITA was expressed in pulmonary interstitial cells rather than endothelial cells. (B) CIITA was also minimally expressed in the ECs of aortic vessels. (C) CIITA could not be enhanced in ECs of ischemic limb. Representative confocal microscopy images of immunofluorescence staining for CIITA (green), CD31 (red), 4’-6-diamidino-2-phenylindole (DAPI, blue). Scar bar, 50 μm. (D) The mRNA level of CIITA was extremely low in HUVECs compared with human myeloid leukemia mononuclear cells (THP-1). Data are mean ± SEM, n = 3 independent experiments. Unpaired Student’s *t*-test, ** $P < 0.01$. (E) The mRNA level of CIITA in VEGFA-165 treated HUVECs remained at a low level. Data are mean ± SEM, n = 3 independent experiments. One-way ANOVA with Bonferroni post-test.

Figure S2. NLRC5 localized to the cytoplasm of LPS-induced ECs. (A) NLRC5 was expressed in the cytoplasm in static ECs of human vessels. Scar bar, 50 μm. (B) *In vivo*, LPS (10 mg/kg, intraperitoneal injection) enhanced the expression of NLRC5 in the cytoplasm of mouse lung endothelial cells (MLECs). Representative confocal microscopy images of immunofluorescence staining for NLRC5 (green), CD31 (red), 4’-6-diamidino-2-phenylindole (DAPI, blue). Scar bar, 20 μm. (C) LPS enhanced the expression of NLRC5 in HUVECs. The protein expression was detected by western blot analysis. (D) The quantifications were normalized by GAPDH. Data are mean ± SEM, n = 3 independent experiments. One-way ANOVA with Bonferroni post-test, ** $P < 0.01$. (E) HUVECs were treated with LPS (100 ng/mL) for 24 h. Nuclear and cytoplasmic fractions were extracted from HUVECs. The protein level of NLRC5 was detected by western blot analysis. (F) Quantification of E. Data are mean

± SEM, n = 3 independent experiments. Unpaired Student's *t*-test, * $P < 0.05$. (G) ICAM-1 and VCAM-1 slightly decreased after siRNA knockdown of NLRC5, which suggested that NLRC5 may inhibit inflammation in HUVECs. Relative mRNA level was quantitated by qPCR. Data are mean ± SEM, n = 4 independent experiments. Unpaired Student's *t*-test, ** $P < 0.01$, # $P < 0.001$.

Figure S3. Decreased NLRC5 had no significant influence on apoptosis in HUVECs. TUNEL Positive cells (green), DAPI (blue), merge (cyan), Scar bar, 50 μm.

Figure S4. Overexpression of NLRC5 rescued tube formation, migration, proliferation of mice lung endothelial cells (MLECs) isolated from NLRC5 global KO mice. (A) Expression of Flag-NLRC5 in AdNC and Ad*NLRC5* transfected MLECs. The protein level was detected by western blot analysis. (B) Quantification of A. Data are mean ± SEM, n = 3 independent experiments. Unpaired Student's *t*-test, ** $P < 0.01$. (C) Overexpressed Flag-NLRC5 translocated into nucleus in VEGFA treated MLECs. Representative confocal microscopy images of immunofluorescence staining for Flag (red), 4'-6-diamidino-2-phenylindole (DAPI, blue). Scar bar, 20 μm. (E) Tube formation of NLRC5 overexpressed MLECs. Scar bar, 50 μm. (D and F) Quantification of branch points and total tube length. Data are mean ± SEM, n = 4 each group. Two-way ANOVA with Bonferroni post-test, *** $P < 0.005$, # $P < 0.001$. (G) Migration of NLRC5 overexpressed MLECs. Scar bar, 50 μm. (H) Quantification of G. Data are mean ± SEM, n = 4 each group. Two-way ANOVA with Bonferroni post-test, * $P < 0.05$. (I) Proliferation of NLRC5 overexpressed MLECs, 5-ethynyl-

2’-deoxyuridine (Edu, red), DAPI (blue). Scar bar, 50 μm. (J) Quantification of I. Data are mean ± SEM, n = 4 each group. Two-way ANOVA with Bonferroni post-test, * $P < 0.05$, # $P < 0.001$.

Figure S5. Establishment and identification of Tie2Cre-NLRC5$^{flox/flox}$ mice. (A) Schematic diagram for establish a line of Tie2Cre-NLRC5$^{flox/flox}$ mice. (B) Identification of NLRC5$^{flox/flox}$ mice by southern blot. (C) The primer used for PCR to identify NLRC5$^{flox/flox}$ genotypes. (D) The decline of NLRC5 mRNA expression in lung tissue of Tie2Cre-NLRC5$^{flox/flox}$ mice. The mRNA level of NLRC5 was detected by qPCR analysis. Data are mean ± SEM, n = 4 independent experiments. Unpaired Student’s *t*-test, * $P < 0.05$.

Figure S6. The retinal angiogenesis of Tie2Cre-NLRC5$^{flox/flox}$ mice. FITC labeled isolectin B4 staining of vascular network in the retinas of P5 pups (green). (A) The vessel length of retina in Tie2Cre-NLRC5$^{flox/flox}$ (CKO) and NLRC5$^{flox/flox}$ (Ctrl) mice. Dashed lines depict the size of retina. Scale bar, 200 μm. (B) Quantification of vessel radial length of retina. Data are mean ± SEM, n = 4 mice/group. Unpaired Student’s *t*-test. (C) The superficial plexus of retina. Scale bar, 50 μm. (D) Quantification of vessel radial length of retina. Data are mean ± SEM, n = 5 mice/group. Unpaired Student’s *t*-test. (E) The sprouting filopodia at the vascular front. The red points indicate the sprout numbers. Scale bar, 50 μm. (F) Quantification of vessel radial length of retina. Data are mean ± SEM, n = 6 mice/group. Unpaired Student’s *t*-test. (G) The expression of NLRC5 in retina of different time points. The protein level was detected by western blot analysis. (H) Quantification of G. Data are mean ± SEM, n = 3 independent

experiments. One-way ANOVA with Bonferroni post-test, ** $P < 0.01$.

Figure S7. Arteriogenesis of Tie2Cre- NLRC5$^{flox/flox}$ mice. (A) Quantification of enlargement of the main artery in ischemic legs in Tie2Cre-NLRC5$^{flox/flox}$ (CKO) and NLRC5$^{flox/flox}$ (Ctrl) mice. Scar bar, 100 μm. (B) Quantification of enlargement of main artery. Enlargement = artery diameter/vessel wall thickness. Data are mean ± SEM, n = 5 mice/group. Two-way ANOVA with Bonferroni post-test, # $P < 0.001$.

Figure S8. Tumor angiogenesis of Tie2Cre-NLRC5$^{flox/flox}$ mice. (A) The body weight curves of Tie2Cre-NLRC5$^{flox/flox}$ (CKO) and NLRC5$^{flox/flox}$ (Ctrl) mice after subcutaneous injection of B16F10 cells. Data are mean ± SEM, n = 6 mice/group. Two-way ANOVA with Bonferroni post-test. (B) The tumor size of Tie2Cre-NLRC5$^{flox/flox}$ and NLRC5$^{flox/flox}$ mice (14 days). (C) The volume curves of Tie2Cre-NLRC5$^{flox/flox}$ and NLRC5$^{flox/flox}$ mice after subcutaneous injection of B16F10 cells. Data are mean ± SEM, n = 6 mice/group. Two-way ANOVA with Bonferroni post-test, # $P < 0.001$. (D) The tumor weight of Tie2Cre-NLRC5$^{flox/flox}$ and NLRC5$^{flox/flox}$ mice (14 days). Data are mean ± SEM, n = 6 mice/group. Two-way ANOVA with Bonferroni post-test, # $P < 0.001$. (E) The CD31-positive cells quantified in tumors. Representative confocal microscopy images of immunofluorescence staining for CD31 (green), 4'-6-diamidino-2-phenylindole (DAPI, blue). Scar bar, 50 μm. (F) Quantification of CD31-positive cells per microscope field. Data are mean ± SEM, n = 6 mice/group, 4 microscope fields/mice. Unpaired Student's *t*-test, *** $P < 0.005$.

Figure S9. The CCD domain of STAT3 was the main domain for the interaction with NLRC5. (A) Plasmid construction of STAT3-FL, △NTD, △CCD, △DBD and △SH2. (B) myc-NLRC5 was co-transfected with STAT3-FL, △NTD, △CCD, △DBD and △SH2, respectively, in HEK293T cells. The lysates were immunoprecipitated and were immunoblotted with antibodies against the indicated proteins.

Figure S10. The STAT3-regulated genes in NLRC5 overexpressed HUVECs. (A-H) The overexpression of NLRC5 induced an increase of STAT3 downstream genes. Relative mRNA level was quantitated by qPCR. Data are mean ± SEM, n = 3 independent experiments. Two-way ANOVA with Bonferroni post-test, * $P < 0.05$, # $P < 0.001$.

Figure S11. STAT3 siRNA impaired the induced enhancement of EC tube formation, migration, and proliferation mediated by overexpression of NLRC5. (A) si*STAT3* inhibited the tube formation in NLRC5 overexpressed HUVECs. Scar bar, 200 μm. (B-C) Quantification of A. Data are mean ± SEM, n = 3 independent experiments. Two-way ANOVA with Bonferroni post-test, * $P < 0.05$, ** $P < 0.01$, *** $P < 0.005$, # $P < 0.001$. (D) siSTAT3 inhibited the migration in NLRC5 overexpressed HUVECs. Scar bar, 200 μm. (E) Quantification of D. Data are mean ± SEM, n = 4 independent experiments. Two-way ANOVA with Bonferroni post-test, * $P < 0.05$, **$P < 0.01$. # $P < 0.001$ (F) STAT3 siRNA inhibited the proliferation in NLRC5 overexpressed HUVECs. 5-ethynyl-2'-deoxyuridine (Edu, red), DAPI (blue). Scar bar, 100 μm. (G) Quantification of F. Data are mean ± SEM, n = 4 independent experiments. Two-way ANOVA with Bonferroni post-test, ** $P < 0.01$. # $P < 0.001$.

Figure S12. Overexpression of STAT3 (WT) rather than STAT3 (Y705F) rescued the decreased angiogenesis in NLRC5 defected endothelial cell line. (A) si*NLRC5* was co-transfected with plasmid of STAT3 (WT) or STAT3 (Y705F) in endothelial cell line (EA.hy926) , and then tube formation assay was performed. Scar bar, 200 μm. (B and C) Quantification of A. Data are mean ± SEM, n = 4 independent experiments. Two-way ANOVA with Bonferroni post-test, ** $P < 0.01$, *** $P < 0.005$. # $P < 0.001$

Figure S13. STAT1 was not a key partner of NLRC5 in VEGFA-165 treated HUVECs. Interferon sensitive response element (ISRE) promoter luciferase reporter plasmid was transfected with myc-NLRC5 plasmid into HEK293T cells for 24 h, cells were treated with PBS or IFN-γ (20 ng/mL) for another 18 h. Promoter activities were normalized to renilla luciferase. The results are expressed as relative luciferase activity. Data are mean ± SEM, n = 3 independent experiments. Two-way ANOVA with Bonferroni post-test, *** $P < 0.005$.

Figure S14. The MHCI related gene after siRNA knockdown of NLRC5 in HUVECs. (A) The efficiency siRNA knockdown of NLRC5 in HUVECs. (B-F) The MHCI related gene moderately declined after siRNA knockdown of NLRC5. Data are mean ± SEM, n = 4 independent experiments. Two-way ANOVA with Bonferroni post-test, * $P < 0.05$, ** $P < 0.01$, # $P < 0.001$.

Figure S1

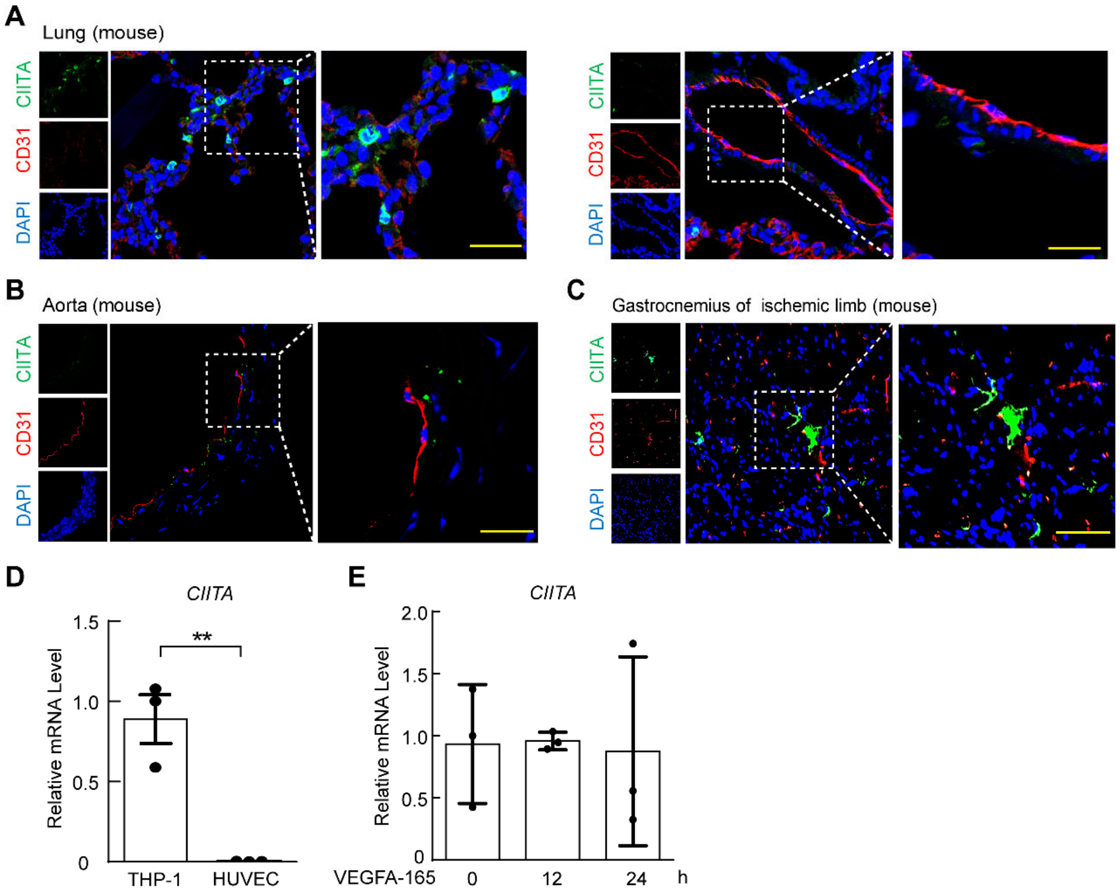

A
Lung (mouse)
CIITA
CD31
DAPI
B
Aorta (mouse)
C
Gastrocnemius of ischemic limb (mouse)
D
CIITA
Relative mRNA Level
1.5
1.0
0.5
0
**
THP-1
HUVEC
E
CIITA
2.0
VEGFA-165
0
12
24
h

Figure S2

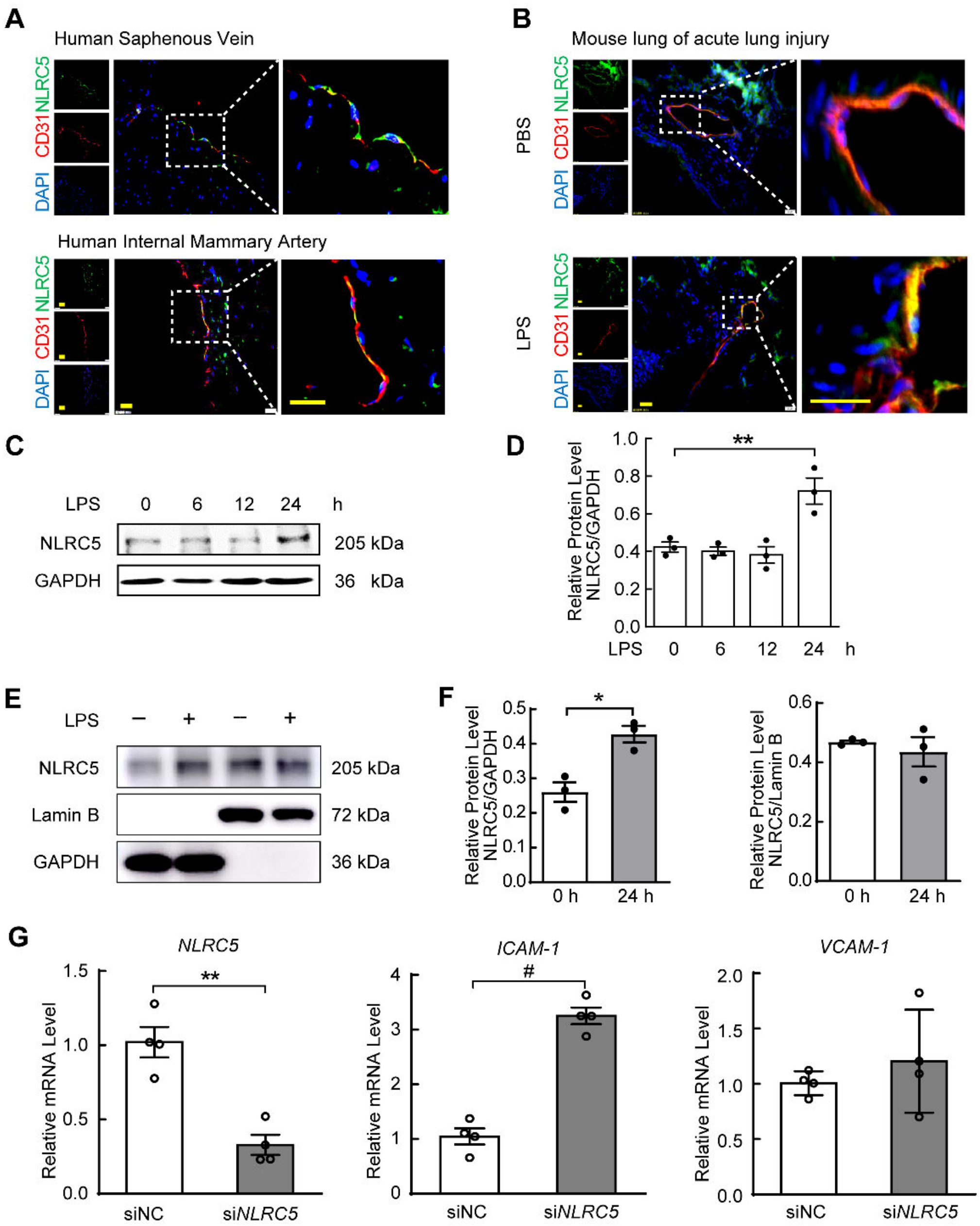

A
Human Saphenous Vein
NLRC5
CD31
DAPI
Human Internal Mammary Artery
B
Mouse lung of acute lung injury
PBS
LPS
C
LPS 0 6 12 24 h
NLRC5 205 kDa
GAPDH 36 kDa
D
Relative Protein Level NLRC5/GAPDH
**
LPS 0 6 12 24 h
E
LPS − + − +
NLRC5 205 kDa
Lamin B 72 kDa
GAPDH 36 kDa
F
Relative Protein Level NLRC5/GAPDH
*
0 h 24 h
Relative Protein Level NLRC5/Lamin B
G
NLRC5
ICAM-1
VCAM-1
Relative mRNA Level
**
#
siNC siNLRC5

Figure S3

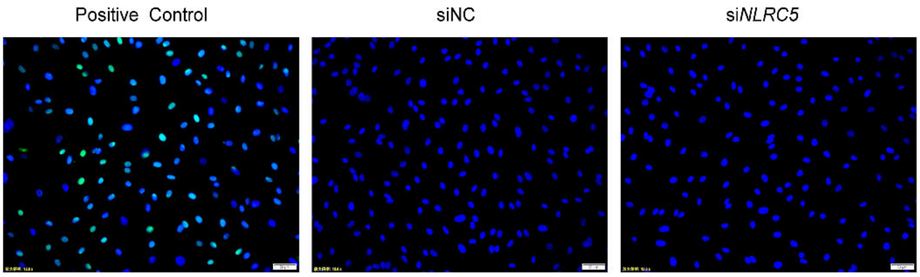

Figure S4

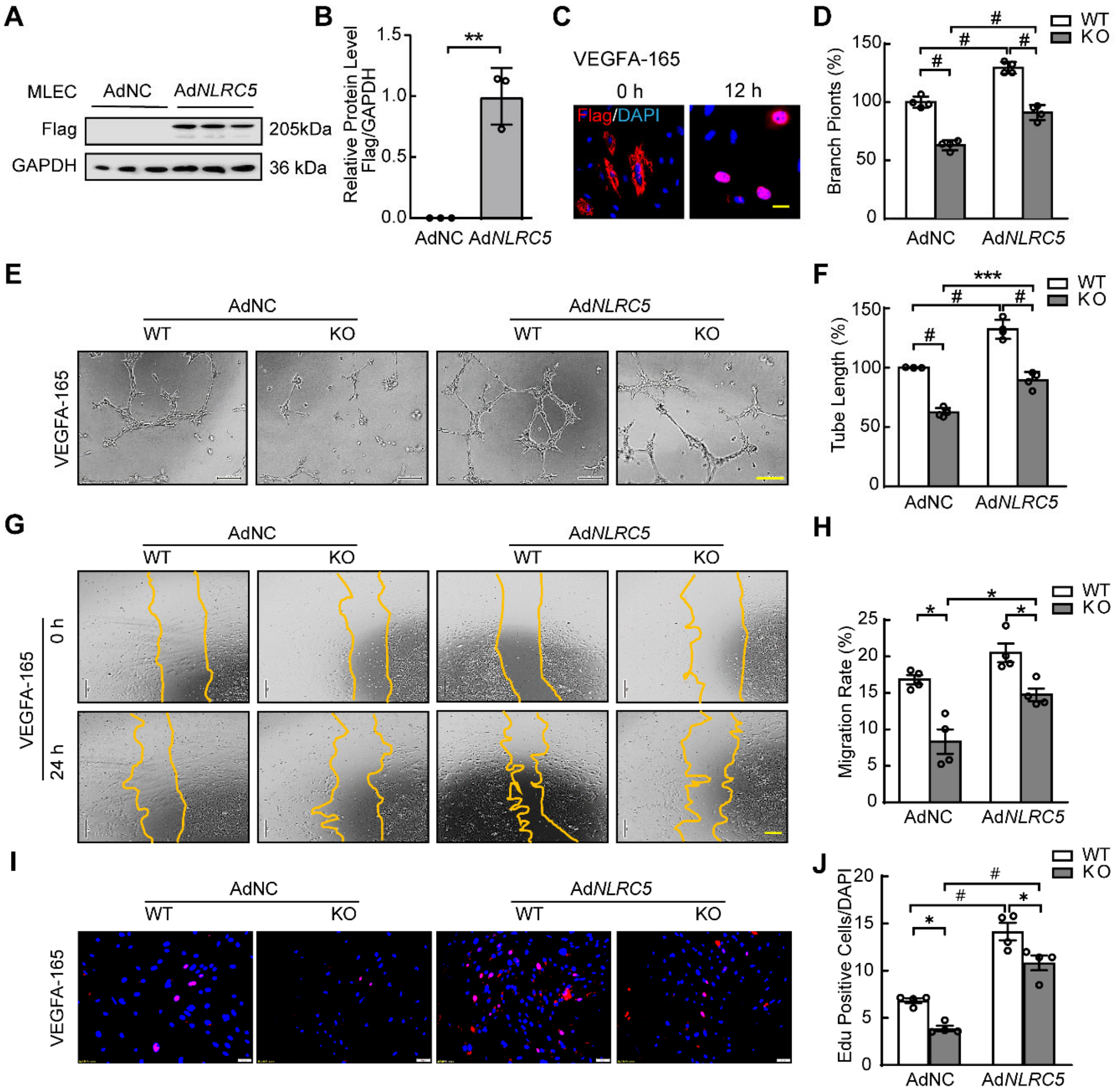

A
MLEC
AdNC
AdNLRC5
Flag
205kDa
GAPDH
36 kDa
B
Relative Protein Level Flag/GAPDH
**
AdNC
AdNLRC5
C
VEGFA-165
0 h
12 h
Flag/DAPI
D
Branch Pionts (%)
WT
KO
#
AdNC
AdNLRC5
E
AdNC
AdNLRC5
WT
KO
VEGFA-165
F
Tube Length (%)
***
#
G
0 h
24 h
H
Migration Rate (%)
*
I
J
Edu Positive Cells/DAPI

Figure S5

A

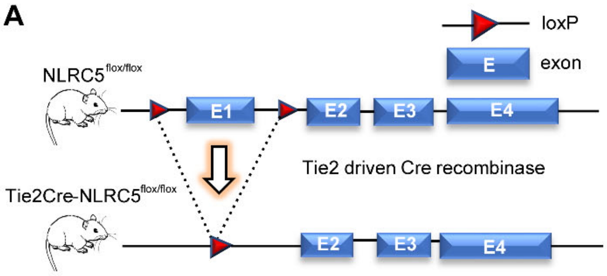

B

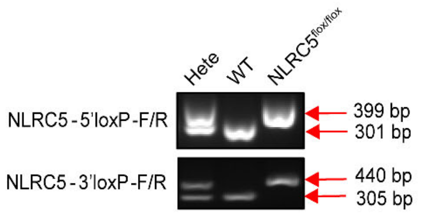

C

Primer	Sequence (5'-3')	Product Size (bp)
NLRC5-5'loxP-F	CTCCTTTCATCTGTCCCCTGATGCC	WT: 301<br>Mut: 399
NLRC5-5'loxP-R	CAGGTCCATGATCCTTAGGCTG	
NLRC5-3'loxP-F	GTAGTCAACATCGCTGCCGAC	WT: 305<br>Mut:440
NLRC5-3'loxP-R	ACACAGTGAGGATGTTTGTCTGCAG	

D

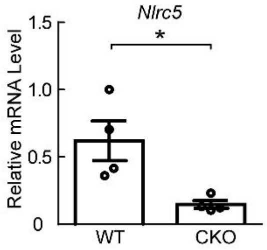

Figure S6

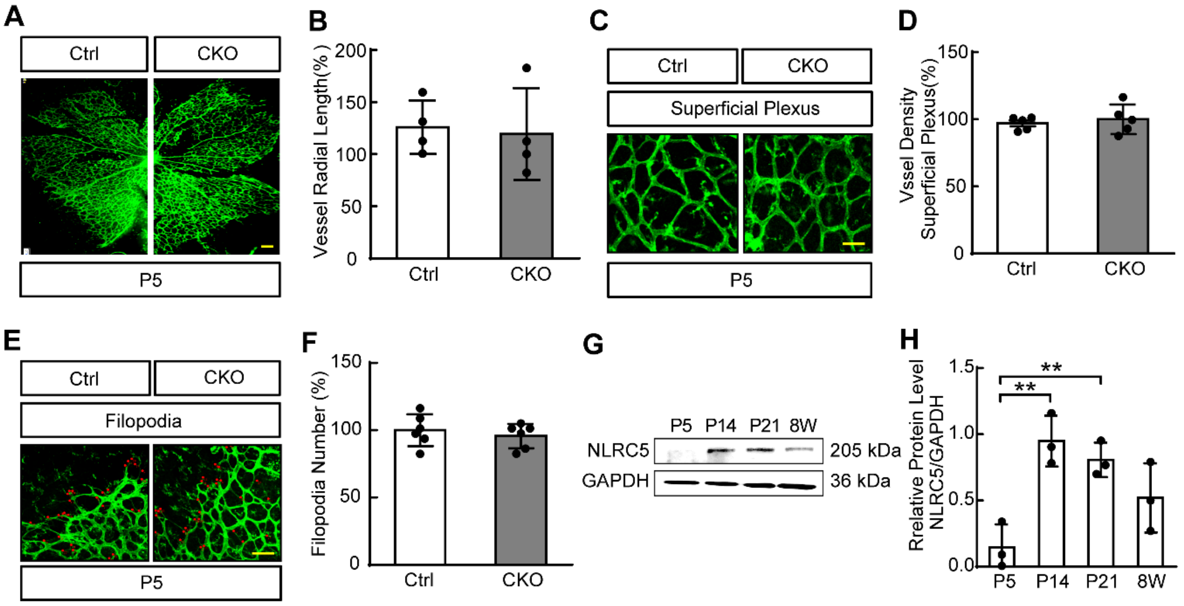
A
Ctrl
CKO
P5
B
Vessel Radial Length(%)
200
150
100
50
0
Ctrl
CKO
C
Ctrl
CKO
Superficial Plexus
P5
D
Vssel Density
Superficial Plexus(%)
150
100
50
0
Ctrl
CKO
E
Ctrl
CKO
Filopodia
P5
F
Filopodia Number (%)
150
100
50
0
Ctrl
CKO
G
P5
P14
P21
8W
NLRC5
GAPDH
205 kDa
36 kDa
H
Rrelative Protein Level
NLRC5/GAPDH
1.5
1.0
0.5
0.0
**
**
P5
P14
P21
8W

Figure S7

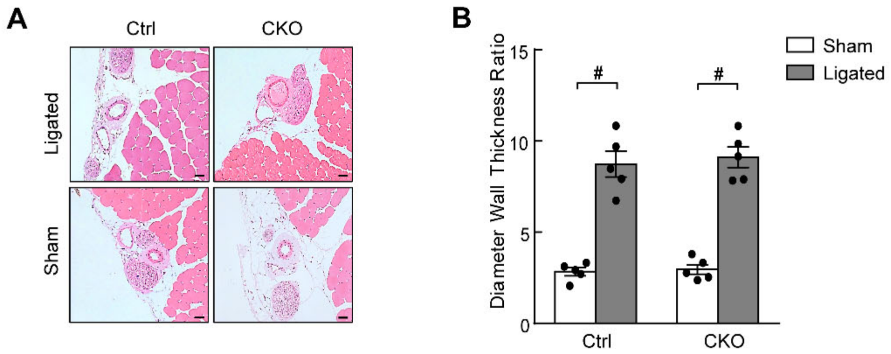
A
Ctrl
CKO
Ligated
Sham
B
Diameter Wall Thickness Ratio
15
10
5
0
#
#
Ctrl
CKO
Sham
Ligated

Figure S8

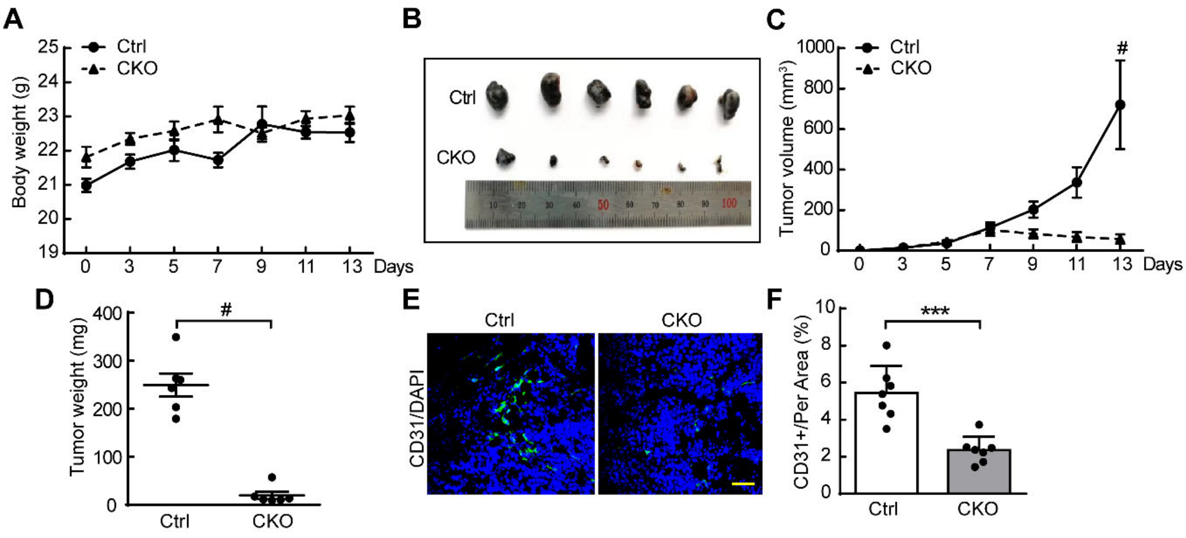
A
Ctrl
CKO
Body weight (g)
25
24
23
22
21
20
19
0
3
5
7
9
11
13
Days
B
Ctrl
CKO
C
Ctrl
CKO
Tumor volume (mm3)
1000
800
600
400
200
0
#
Days
D
Tumor weight (mg)
400
300
200
100
0
#
Ctrl
CKO
E
Ctrl
CKO
CD31/DAPI
F
CD31+/Per Area (%)
10
8
6
4
2
0
***
Ctrl
CKO

Figure S9

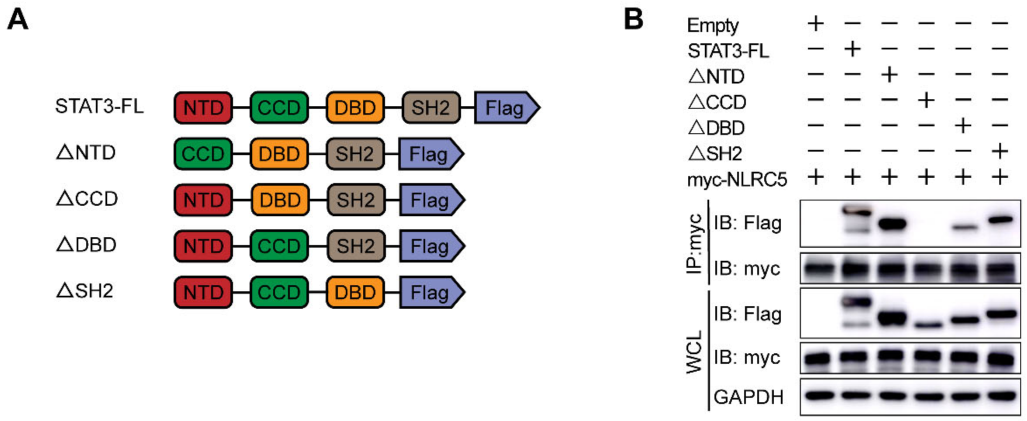
A
STAT3-FL
NTD
CCD
DBD
SH2
Flag
△NTD
△CCD
△DBD
△SH2
B
Empty
STAT3-FL
△NTD
△CCD
△DBD
△SH2
myc-NLRC5
IP:myc
IB: Flag
IB: myc
WCL
IB: Flag
IB: myc
GAPDH

Figure S10

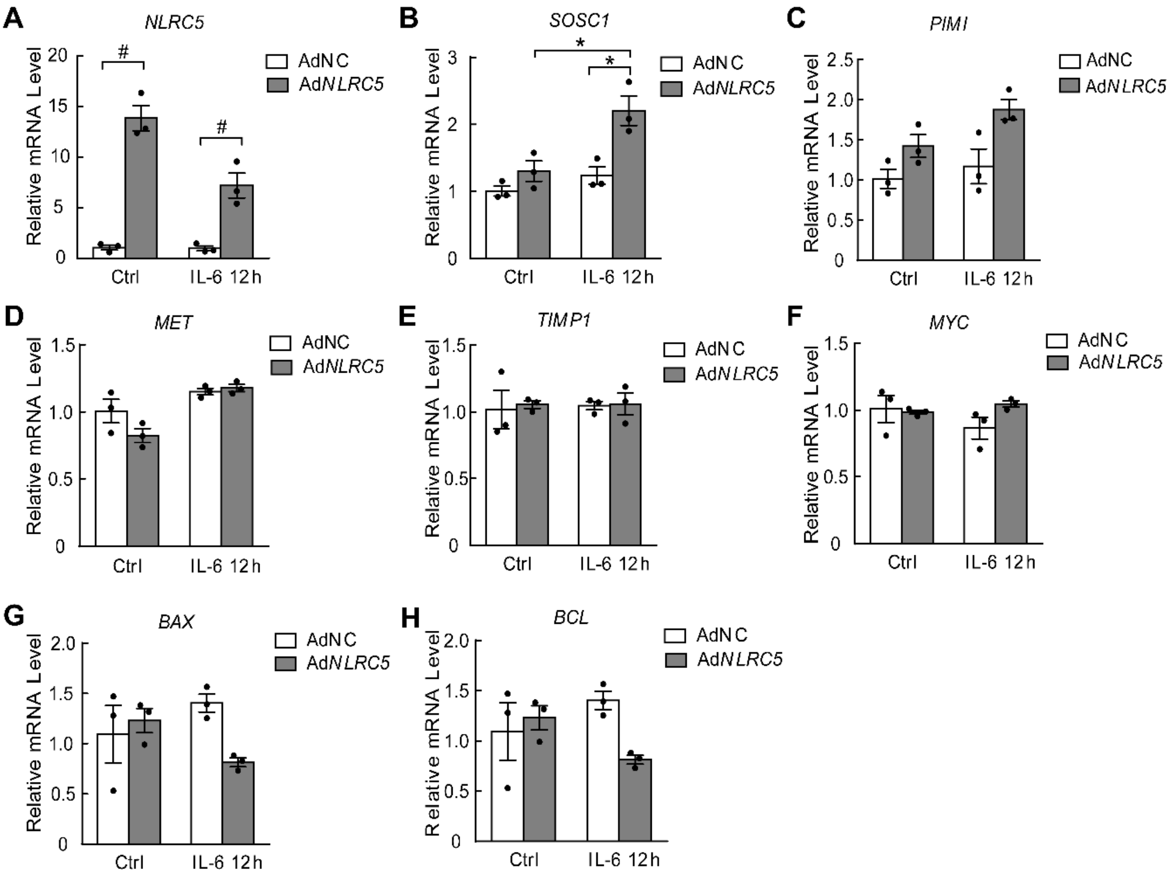

A
NLRC5
Relative mRNA Level
20
15
10
5
0
#
#
AdNC
AdNLRC5
Ctrl
IL-6 12h
B
SOSC1
Relative mRNA Level
3
2
1
0
*
*
AdNC
AdNLRC5
Ctrl
IL-6 12h
C
PIMI
Relative mRNA Level
2.5
2.0
1.5
1.0
0.5
0
AdNC
AdNLRC5
Ctrl
IL-6 12h
D
MET
Relative mRNA Level
1.5
1.0
0.5
0
AdNC
AdNLRC5
Ctrl
IL-6 12h
E
TIMP1
Relative mRNA Level
1.5
1.0
0.5
0
AdNC
AdNLRC5
Ctrl
IL-6 12h
F
MYC
Relative mRNA Level
1.5
1.0
0.5
0
AdNC
AdNLRC5
Ctrl
IL-6 12h
G
BAX
Relative mRNA Level
2.0
1.5
1.0
0.5
0
AdNC
AdNLRC5
Ctrl
IL-6 12h
H
BCL
Relative mRNA Level
2.0
1.5
1.0
0.5
0
AdNC
AdNLRC5
Ctrl
IL-6 12h

Figure S11

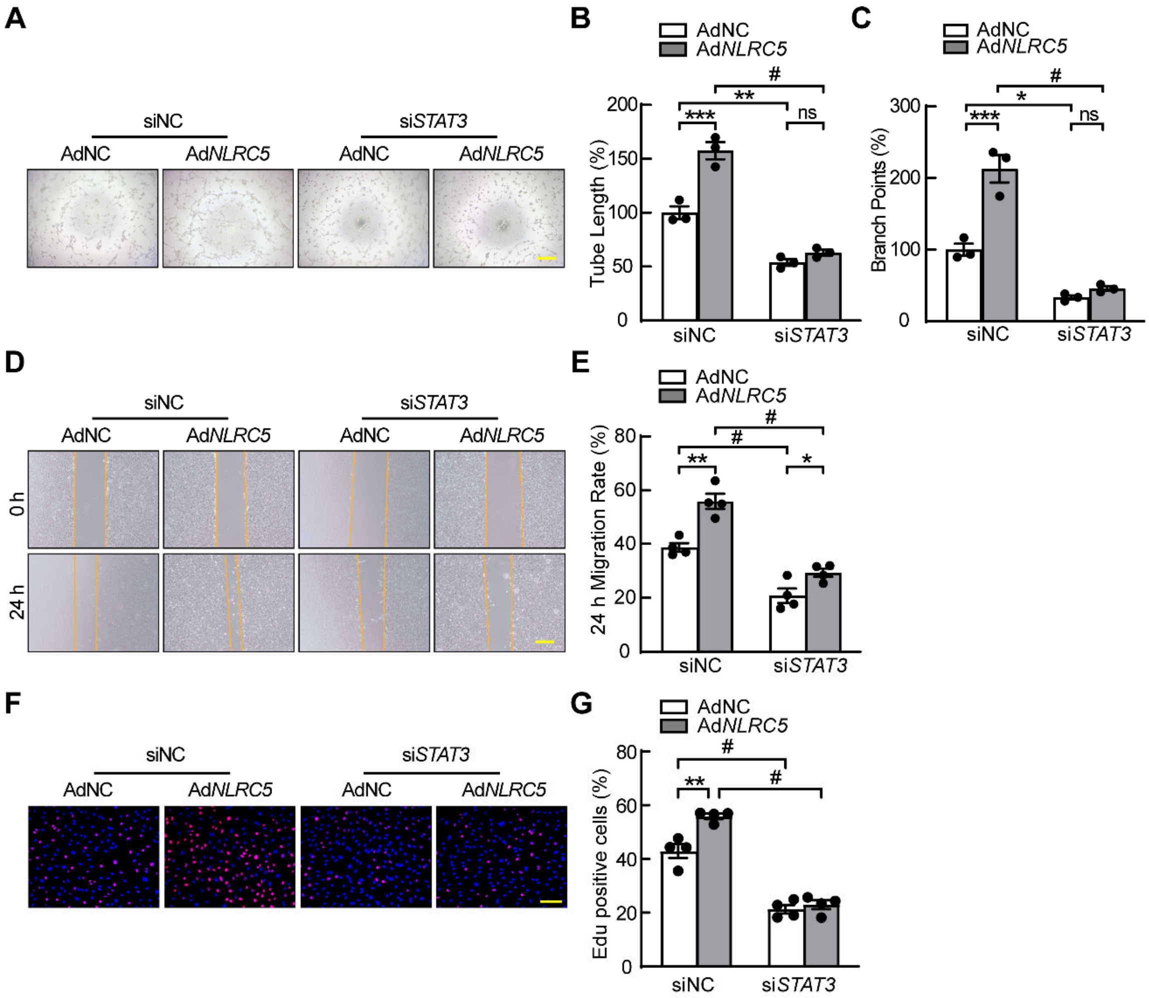
A
siNC
siSTAT3
AdNC
AdNLRC5
AdNC
AdNLRC5
B
AdNC
AdNLRC5
Tube Length (%)
siNC
siSTAT3
C
AdNC
AdNLRC5
Branch Points (%)
siNC
siSTAT3
D
siNC
siSTAT3
AdNC
AdNLRC5
AdNC
AdNLRC5
0 h
24 h
E
AdNC
AdNLRC5
24 h Migration Rate (%)
siNC
siSTAT3
F
siNC
siSTAT3
AdNC
AdNLRC5
AdNC
AdNLRC5
G
AdNC
AdNLRC5
Edu positive cells (%)
siNC
siSTAT3

Figure S12

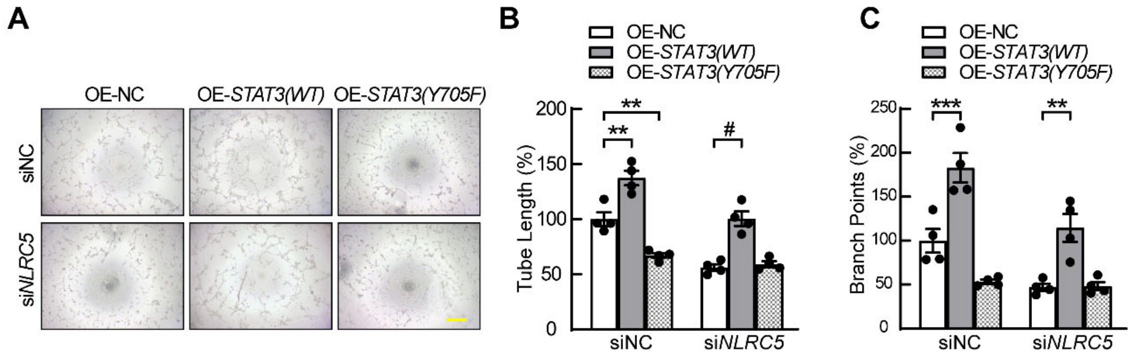
A
OE-NC
OE-STAT3(WT)
OE-STAT3(Y705F)
siNC
siNLRC5
B
OE-NC
OE-STAT3(WT)
OE-STAT3(Y705F)
Tube Length (%)
200
150
100
50
0
**
**
#
siNC
siNLRC5
C
OE-NC
OE-STAT3(WT)
OE-STAT3(Y705F)
Branch Points (%)
250
200
150
100
50
0
***
**
siNC
siNLRC5

Figure S13

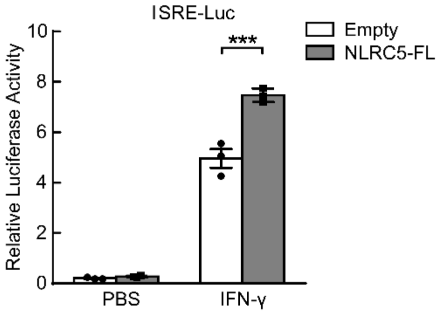

ISRE-Luc
Empty
NLRC5-FL
***
10
8
6
4
2
0
Relative Luciferase Activity
PBS
IFN-γ

Figure S14

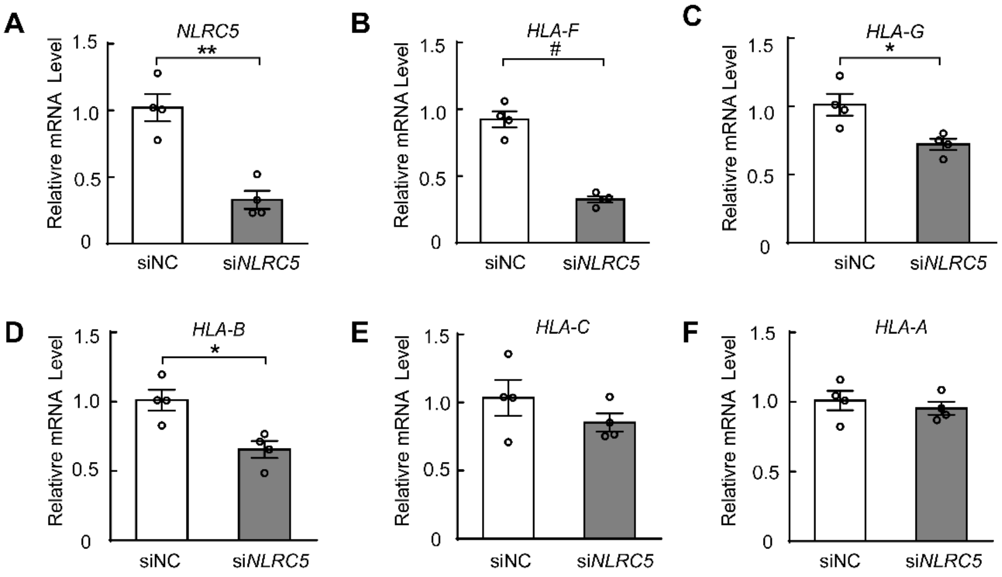
A
NLRC5
**
Relativre mRNA Level
1.5
1.0
0.5
0
siNC
siNLRC5
B
HLA-F
#
Relativre mRNA Level
1.5
1.0
0.5
0
siNC
siNLRC5
C
HLA-G
*
Relativre mRNA Level
1.5
1.0
0.5
0
siNC
siNLRC5
D
HLA-B
*
Relativre mRNA Level
1.5
1.0
0.5
0
siNC
siNLRC5
E
HLA-C
Relativre mRNA Level
1.5
1.0
0.5
0
siNC
siNLRC5
F
HLA-A
Relativre mRNA Level
1.5
1.0
0.5
0
siNC
siNLRC5

Table 1.1. The list of antibodies

Antibody	Catalogue	Dilution Ratio	Application
NLRC5	Millipore, Cat# MABF260	1:300	WB
NLRC5	Abcam, Cat# ab105411	1:100	IF
CD31 (human)	R&D system, Cat# BBA7	1:100	IF
CD31 (mouse)	BD Biosciences, Cat# 550274	1:100	IF
CD45	R&D system, Cat# AF114	1:50	IF
CD144	BD Biosciences, Cat# 555289	1:100	IF
Ki67	Abcam, Cat# AF7649	1:50	IF
STAT3	CST, Cat# 9139	1:1000	WB
		1:50	IP
		1:50	IF
		1:10	ChIP
Phospho-STAT3	CST, Cat# 9145	1:1000	WB
(Tyr705)			
eNOS	BD Biosciences, Cat# 610297	1:1000	WB
Phospho-eNOS	Santa Cruz, Cat# sc-21871-R	1:500	WB
(Ser1177)			
Phospho-AKT	CST, Cat# 4058	1:1000	WB
(Ser473)			
AKT	CST, Cat# 4691	1:1000	WB
STAT1	CST, Cat# 14994	1:1000	WB
PPARγ	Santa Cruz, Cat# sc-7273	1:500	WB
		1:100	IP
Myc-tag	CST, Cat# 2276	1:2000	WB
		1:50	IP
Flag	CST, Cat# 14793	1:1000	WB
		1:50	IP
		1:50	ChIP
Lamin B	Boster, Cat# BA1228	1:400	WB
GAPDH	CST, Cat# 5174	1:5000	WB
APC-CD31	BD Biosciences, Cat# 551262	1:100	FASC
PerCP-CD45	BD Biosciences, Cat# 557235	1:100	FASC
PE-CD11b	BD Biosciences, Cat# 557397	1:100	FASC
APC-Gr1	BD Biosciences, Cat# 553129	1:100	FASC

Table 1.2. The list of antibodies

Antibody	Catalogue	Dilution Ratio	Application
HRP conjugated Goat Anti-Rabbit IgG (H+L)	Jackson Immuno Research, Cat# 115-036-003	1:5000	WB
HRP conjugated Goat Anti-Rabbit IgG (H+L)	Jackson Immuno Research, Cat# 111-036-003	1:5000	WB
HRP conjugated Goat Anti-Rat IgG (H+L)	Jackson Immuno Research, Cat# 112-036-003	1:5000	WB
Donkey anti-Rabbit IgG (H+L), Alexa Fluor Plus 594	Invitrogen, Cat# A32754	1:100	ICC, IF
Donkey anti-mouse IgG (H+L), Alexa Fluor Plus 594	Invitrogen, Cat# 32744	1:100	ICC, IF
Donkey anti-Rabbit IgG (H+L), Alexa Fluor Plus 488	Invitrogen, Cat# 32731	1:100	ICC, IF
Donkey anti-mouse IgG (H+L), Alexa Fluor Plus 488	Invitrogen, Cat# 32723	1:100	ICC, IF
Donkey anti-Rabbit IgG (H+L), Alexa Fluor Plus 488	Invitrogen, Cat# 32723	1:100	ICC, IF
Donkey anti-Rabbit IgG (H+L), Alexa Fluor Plus 647	Invitrogen, Cat# 32733	1:100	ICC, IF
Donkey Anti-Sheep IgG H&L (Alexa Fluor® 488)	Abcam Cat# ab150177	1:100	ICC, IF

Table 2. qPCR primer sequence, ChIP-PCR primer sequence, PCR primer sequence, siRNA sequence

Primer	Sequence	Application
NLRC5 (human)	Forward: 5'-GCTCGGCAACAAGAACCTGT-3'<br>Reverse: 5'-GTCCAAGGTCTCGTTCCT-3'	qPCR
HLA-A (human)	Forward: 5'-AAAAGGAGGGAGTTACACTCAGG-3'<br>Reverse: 5'-GCTGTGAGGGACACATCAGAG-3'	qPCR
HLA-B (human)	Forward: 5'-CAGTTCGTGAGGTTCGACAG-3'<br>Reverse: 5'-CAGCCGTACATGCTCTGGA-3'	qPCR
HLA-F (human)	Forward: 5'-TGGCCCTGACCGATACTTG-3'<br>Reverse: 5'-GCAGGAATTGCGTGTCGTC-3'	qPCR
CIITA (human)	Forward: 5'-CCTGGAGCTTCTTAACAGCGA-3'<br>Reverse: 5'-TGTGTCGGGTTCTGAGTAGAG-3'	qPCR
VCAM-1 (human)	Forward: 5'-GGGAAGATGGTCGTGATCCTT-3'<br>Reverse: 5'-TCTGGGGTGGTCTCGATTTTA-3'	qPCR
ICAM-1 (human)	Forward: 5'-ATGCCCAGACATCTGTGTCC-3'<br>Reverse: 5'-ATGCCCAGACATCTGTGTCC-3'	qPCR
CCND1 (human)	Forward: 5'-GCTGCGAAGTGGAAACCATC-3'<br>Reverse: 5'-CCTCCTTCTGCACACATTTGAA-3'	qPCR
PIM1 (human)	Forward: 5'-ACGAAAACATCCTTATCGACCT-'3<br>Reverse: 5'-CGTCATGCTCGAAAGGAATATC-'3	qPCR
SOCS1 (human)	Forward: 5'-GAACTGCTTTTTCGCCCTTAG-3'<br>Reverse: 5'-GAAGAGGCAGTCGAAGCTC-3'	qPCR
ANGPT2 (human)	Forward: 5'-AACTTTCGGAAGAGCATGGAC-3'<br>Reverse: 5'-CGAGTCATCGTATTCGAGCGG-3'	qPCR
GAPDH (human)	Forward: 5'-GGAGCGAGATCCCTCCAAAAT-3'<br>Reverse: 5'-GGCTGTTGTCATACTTCTCATGG-3	qPCR
CCND1 (human)	Forward: 5'-CTCTGCCGGGCTTTGATCTT-3'<br>Reverse: 5'-ATGGTTTCCACTTCGCAGCA-3'	ChIP
ANGPT2 (human)	Forward: 5'-TCTTTAATTGGTTCCCTTAG-3'<br>Reverse: 5'-TAGGCTTCACCAGACAACC-3'	ChIP
Global KO NLRC5 (mouse)	Forward: 5'-CTGCCCAGGGAATTATGCTA-3'<br>Reverse: 5'-AATGTGTGCGAGGCCAGAG-3'	PCR
Global WT NLRC5 (mouse)	Forward: 5'-CAGTCCATCACCTGGGAAGT-3'<br>Reverse: 5'-ATCCTGTGCTGCTCCTCAGT-3'	PCR

siSTAT3 (human)	Forward: 5'-CCCGGAAAUUUAACAUUCU-3'<br>Reverse: 5'-AGAAUGUUAAAUUUCCGGG-3'	siRNA
